# Mapping the Role of Monomer Conformation in the Amyloid Formation of α‑Synuclein Splice Variants

**DOI:** 10.1021/jacs.5c12366

**Published:** 2025-10-24

**Authors:** Katherine M. Dewison, Alexander I. P. Taylor, David J. Brockwell, Sheena E. Radford

**Affiliations:** Astbury Centre for Structural Molecular Biology and School of Molecular and Cellular Biology, Faculty of Biological Sciences, 4468 University of Leeds, Leeds LS2 9JT, U.K.

## Abstract

Amyloid formation of the protein α-synuclein (αSyn) is a hallmark of pathogenesis in Parkinson’s disease, multiple system atrophy, and dementia with Lewy bodies. Research has predominantly focused on the 140-amino acid αSyn sequence, yet the *SNCA* gene can be alternatively spliced to generate several different isoforms, including αSynΔ3, αSynΔ5, and αSynΔ3Δ5. Here, we have used experimental and computational approaches to characterize these splice variants, in addition to the full-length αSyn, in terms of their monomer conformation and amyloid propensity as a function of changes in ionic strength. Kinetic analysis of amyloid formation, flow-induced dispersion analysis, and coarse-grained molecular dynamics simulations reveal a striking correlation between monomer conformation and the rate of secondary nucleation of amyloid formation, and we show that this is governed by both global conformation of the polypeptide chain and local contacts in the hydrophobic core domain and acidic C-terminal domain. By combining changes in amino acid sequences and ionic strength, our analysis reveals the importance of local contacts and long-range electrostatic interactions in driving the kinetics of amyloid formation of αSyn.

## Graphical abstract

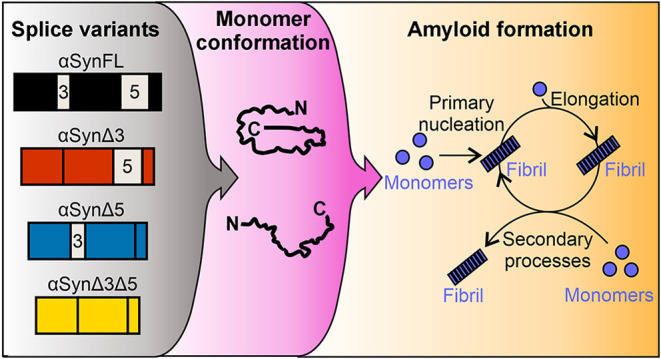

## Introduction

Parkinson’s disease (PD), multiple system atrophy (MSA), and dementia with Lewy bodies (DLB) are conditions categorized as ‘synucleinopathies’, which exhibit hallmark intracellular aggregates of α-synuclein (αSyn).^1^ Given the prominence of these amyloid aggregates in synucleinopathies, they are hypothesized to play a role in disease,^2–4^ and are key markers of pathogenesis. Despite many efforts to develop therapeutics that target αSyn aggregation, none have been successful to date,^5^ highlighting that a better understanding of the molecular mechanisms underlying the aggregation of αSyn into amyloid and the etiology of synucleinopathies is needed. Attempts to develop this understanding have included studying the role of familial disease variants,^6,7^ disease-relevant truncations,^8–10^ membranes,^11–16^ variants that have been designed based on *in silico* predictions of aggregation,^17–19^ and the addition of chaperones,^20,21^ nanobodies,^22,23^ and other αSyn-binding peptides^24^ in the mechanism of amyloid formation.

The full-length 140-amino acid αSyn protein is comprised of three domains: an amphipathic N-terminal domain (residues 1–60) that is required for membrane-binding^25,26^ and facilitates the physiological function of αSyn in membrane vesicle fusion at the synapse;^27–29^ the hydrophobic core domain (residues 61–95, commonly referred to as the nonamyloid β component (NAC)^30^), which is necessary and sufficient to form amyloid;^31^ and the highly acidic C-terminal domain (residues 96–140), which binds metal ions,^32,33^ functions in SNARE complex assembly,^34^ and is thought to protect αSyn from amyloid formation.^8,9^ The three biochemically distinct regions of αSyn, which together facilitate functional ‘promiscuity’,^35^ result in a protein with a polarized sequence that is prone to self-assemble into amyloid fibrils.

The role of sequence length variation in the self-assembly and disease-context of a number of amyloidogenic proteins has been widely investigated hitherto, these include examples such as the 3R and 4R splice variants of tau being implicated in different diseases,^36^ the processing of amyloid-β to between 39 and 43 amino acids controlling its amyloidogenicity and involvement in disease,^37^ and naturally occurring C-terminal truncations of αSyn dramatically accelerating the rate of amyloid formation.^8,9,38^ Although most research characterizing the mechanisms of αSyn amyloid formation have focused on the 140-amino acid sequence, the gene encoding αSyn, *SNCA*, can be spliced to generate at least three alternative isoforms^39,40^ (Figure 1). Specifically, exon 3 (encoding residues 41 to 54) and exon 5 (encoding residues 103 to 130) can be spliced in or out of the final transcript to generate either full-length αSyn (αSynFL, which contains residues encoded by both exons 3 and 5) or αSynΔ3, αSynΔ5, and αSynΔ3Δ5. The expression levels of the alternative splice variants across different brain regions and diseases (as revealed by mRNA levels) have been characterized in several publications^41–46^ (Table S1). While some of these results indicate that the expression levels are changed in disease, others report similar levels between disease and control tissue samples. Furthermore, it should be noted that the presence of these variants in the pathological lesions of synucleinopathies (Lewy bodies or glial cytoplasmic inclusions) has not yet been reported, and their impact in disease etiology remains to be determined. Despite this, recent work has demonstrated that these variants display distinct aggregation propensities^47–49^ and MSA-associated single nucleotide polymorphisms in the noncoding regions of the *SNCA* gene lead to upregulation of αSynΔ5.^50^ Along with evidence that this variant is indeed translated in the brain,^51^ this highlights the importance of better understanding of the amyloid formation mechanisms of these variants and their roles in the pathogenesis of synucleinopathies.

**Figure 1:**
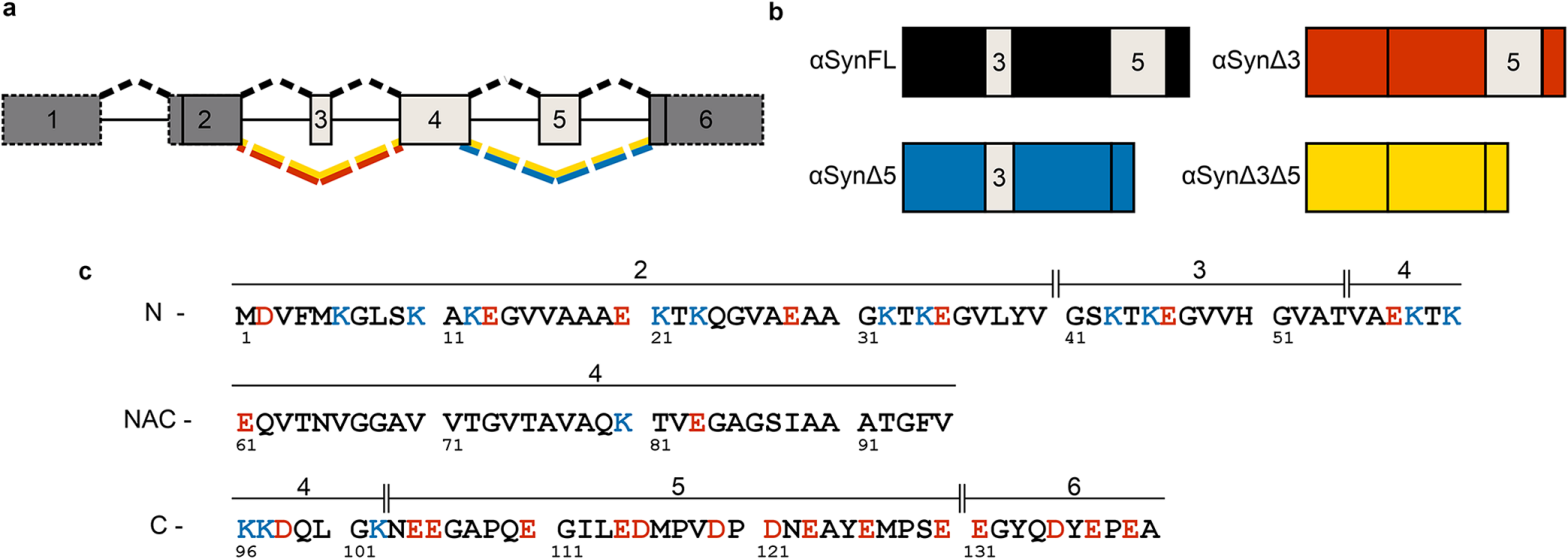
Alternative splice variants of αSyn used in this work. (a) Schematic representation of *SNCA*. The boxes represent exons (1 to 6) and the solid lines represent introns. Dark gray boxes are constitutively spliced in, but light gray boxes are cassette exons. Boxes surrounded by dashed lines show exons that are not translated, and boxes with solid lines show exons that are translated. αSynFL is generated by the splicing path indicated by the black dashed lines above the exons/introns. Alternative splicing is achieved by exon skipping (shown by colored dashed lines) of either exon 3 (red), exon 5 (blue), or both exons 3 and 5 (yellow). Note that although an additional exon has been identified in the 5′ untranslated region of *SNCA* (NCBI accession number NG_011851), previous publications have continued to refer to the exons as outlined in (a), so we have upheld this nomenclature for ease of comparison. (b) Representation of the proteins generated from the alternative splicing of *SNCA*. Where cassette exons are included in the protein, they are labeled with their exon number. (c) Amino acid sequence of αSyn, with the corresponding exons in which they are encoded displayed above, and the domain indicated to the left (N=N-terminal domain, NAC = hydrophobic core domain, C=C-terminal domain). Negatively charged residues are colored in red, and positively charged residues are colored in blue.

Here, we set out to investigate how the monomer conformation of αSyn splice variants influences their amyloidogenicity. Given the distinct sequence patterning of the three domains of αSyn (Figure 1c), and the fact that αSynFL is known to form transient long-range intramolecular electrostatic interactions in its monomeric state^52–55^ that must be significantly perturbed by deletion of residues encoded by exons 3 and/or 5, we examined the effect of ionic strength on the rate and mechanisms of amyloid formation of the different variant sequences. Supported by global conformational measurements using flow-induced dispersion analysis (FIDA)^56^ and coarse-grained molecular dynamics (MD) simulations using the CALVADOS 2 force field,^57,58^ we reveal a striking correlation between monomer conformation and the rate of amyloid formation that is consistent across all splice variants and ionic strengths. Our analysis shows that changes in the global conformations of αSyn are coupled to changes in local contacts, and furthermore, that interactions within the NAC and C-terminal regions critically alter the rate of secondary nucleation of amyloid formation. Together, these features rationalize the strikingly different amyloid propensities of the splice variants.

## Results

### Residues Encoded by Exons 3 and 5 of *SNCA* have Distinct Effects on Amyloid Formation

To investigate the amyloid potential of the alternative splice variants of αSyn, we generated recombinant proteins in which the amino acid sequences corresponding to exon 3 (^41^GSKTKEGVVHGVAT^54^) and/or exon 5 (^103^NEEGAPQEGILEDMPVDPDNEAYEMPSE^130^) are deleted (Figure 1). The rate of amyloid formation for each of these variants was then monitored using thioflavin T (ThT) fluorescence at starting monomer concentrations ranging from 20 to 100 μM (Figure 2a). In accord with previous findings,^47^ the time to reach half the maximal fluorescence (*T*_50_, Experimental Section and Table S2) of αSynFL and αSynΔ3 are similar and concentration-dependent over this range (Figure 2b), and we find that the calculated scaling exponents (the gradient of the log *T*_50_ vs log [αSyn], see Experimental Section and eq 1) are −0.55 and −0.49, respectively (Table S3). We then used the fitting platform AmyloFit to compare four different models of amyloid assembly for these two variants: elongation-dominated, secondary nucleation-dominated, fragmentation-dominated, and multistep secondary nucleation-dominated assembly^59^ (Figure S1). For αSynFL, secondary nucleation, fragmentation, and multistep secondary nucleation-dominated models achieved similarly good fits. For αSynΔ3, the model comparison favored multistep secondary nucleation. By contrast, αSynΔ5 and αSynΔ3Δ5 form amyloid more rapidly than αSynFL and αSynΔ3 and exhibited only minor changes in the rate of amyloid formation across the range of concentrations used (Figure 2a,b and Table S2), such that the scaling exponents for these variants is closer to zero (γ = 0.17 and −0.064 for αSynΔ5 and αSynΔ3Δ5, respectively (Table S3)) and for αSynΔ5 this concentration-independence is also observed at lower monomer concentrations (from 2.5 to 20 μM (Figure S2)). The lack of concentration-dependence of the rate of amyloid formation of αSynΔ5 and αSynΔ3Δ5 suggests saturation of primary and/or secondary nucleation, such that conformational conversion from the fibril-bound monomer to the fibrillar state, rather than binding to catalytic sites, is the rate-determining step. For all variants, insoluble fibrillar material was formed at the end of the reaction as judged by a pelleting assay and negative stain EM (Figure 2c,d and Table S5).

**Figure 2:**
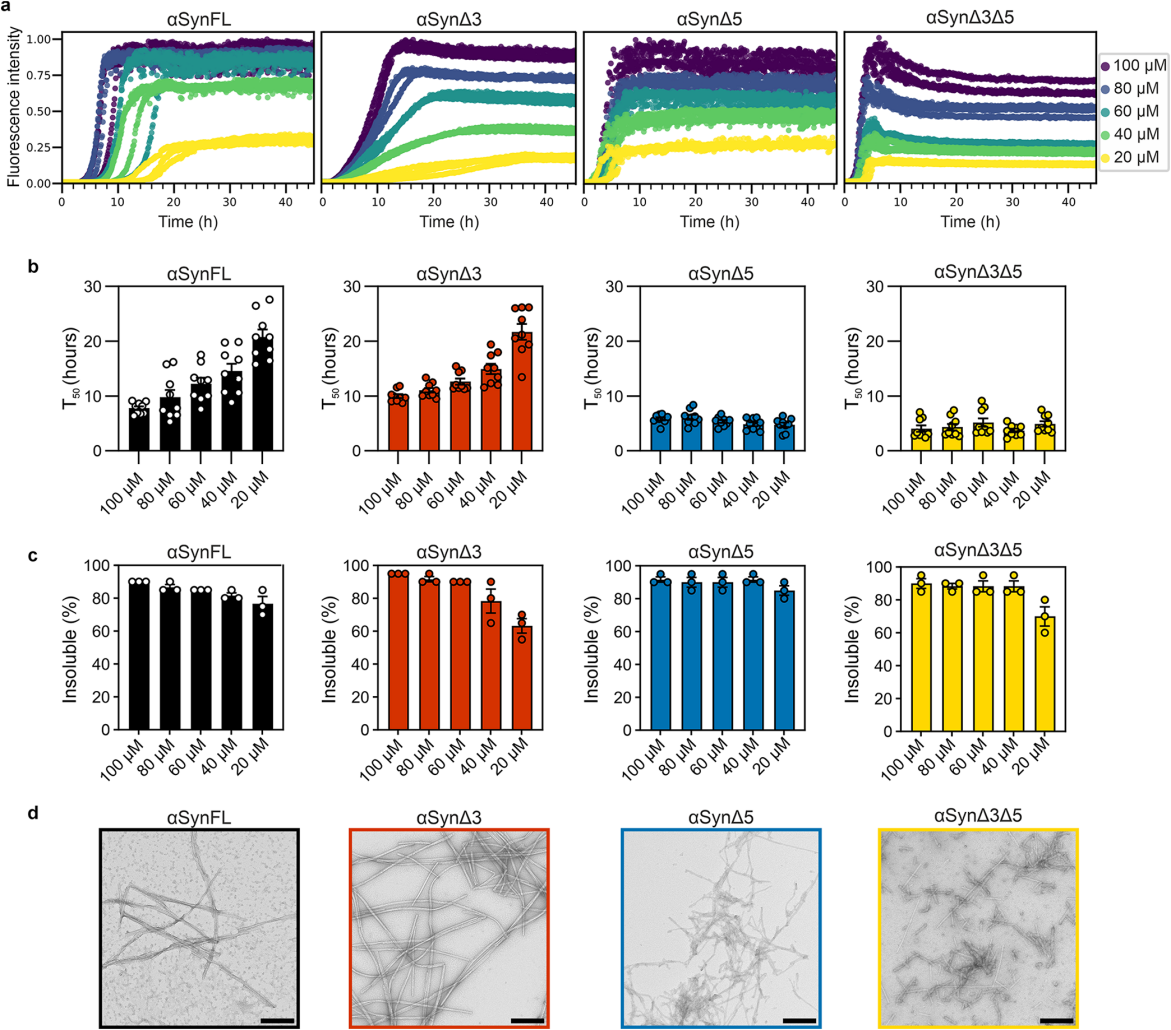
*De novo* fibril formation at different protein concentrations for the alternative splice variants of αSyn. (a) ThT fluorescence versus time for each of the variants (variant name indicated at the top of the plot) at monomer concentrations ranging from 20 to 100 μM (see key) in PBS (137 mM NaCl, 2.7 mM KCl, 8.1 mM Na_2_HPO_4_, and 1.5 mM KH_2_PO_4_; pH 7.4). Data are normalized to the maximum fluorescence intensity of each variant at 100 μM protein concentration. (b) Time to reach 50% of the maximum fluorescence (*T*_50_) for each condition. Individual values from three repeats, each containing three replicates, are plotted. The mean is represented by the bar height, and the error bars show the standard error of the mean (SEM). (c) Percent insoluble material at the end point of ThT reactions as quantified using a pelleting assay (Experimental Section), where each data point corresponds to a ThT repeat, and the error bar is SEM (d) Negative stain transmission electron microscopy (TEM) images of the material formed at the end of the ThT assay for each variant. Scale bar, 250 nm.

We next investigated the capacity of the alternative splice variants to cross-seed the assembly of the αSynFL monomer into amyloid. The justification for this is that the *SNCA* mRNA isoform encoding αSynFL constitutes ∼95–97% of all *SNCA* mRNA transcripts that are expressed in the brain,^60^ but whether the lowly populated splice variants, particularly the more amyloidogenic αSynΔ5 and αSynΔ3Δ5, are capable of triggering conversion of αSynFL into amyloid under the conditions of these experiments was unknown. To test this, amyloid fibrils formed at the end of the *de novo* self-assembly reactions of each variant (at 100 μM protein concentration) were collected and sonicated to generate short fibril seeds (Experimental Section). The preformed fibril fragments were then added to αSynFL monomers at a fibril seed concentration of 10% (*v/v*) (monomer equivalent), and ThT fluorescence was used to monitor fibril formation (quiescent conditions, wherein elongation is the dominant mechanism of seeded growth^61^). As a control, self-seeding experiments (where the identity of the fibril seed was the same as that of the added monomer) were performed to show that the added fibrils could recruit their own monomer (Figure S3). Remarkably, and consistent with previous findings,^49^ the only cross-seeding reaction that resulted in a significant increase in ThT fluorescence and the presence of fibrils at the end of the reaction was αSynFL monomer in the presence of αSynΔ5 fibril seeds (Figure 3 and Table S6). Hence, the residues encoded by exon 3 (^41^GSKTKEGVVHGVAT^54^) are required for the αSyn variants to cross-seed fibril formation of the full-length protein monomers. These residues form part of the fibril core in many of the resolved αSyn amyloid structures,^62,63^ potentially rationalizing why αSynFL is unable to adopt the same amyloid fold as αSynΔ3 or αSynΔ3Δ5 in the process of elongation.

**Figure 3:**
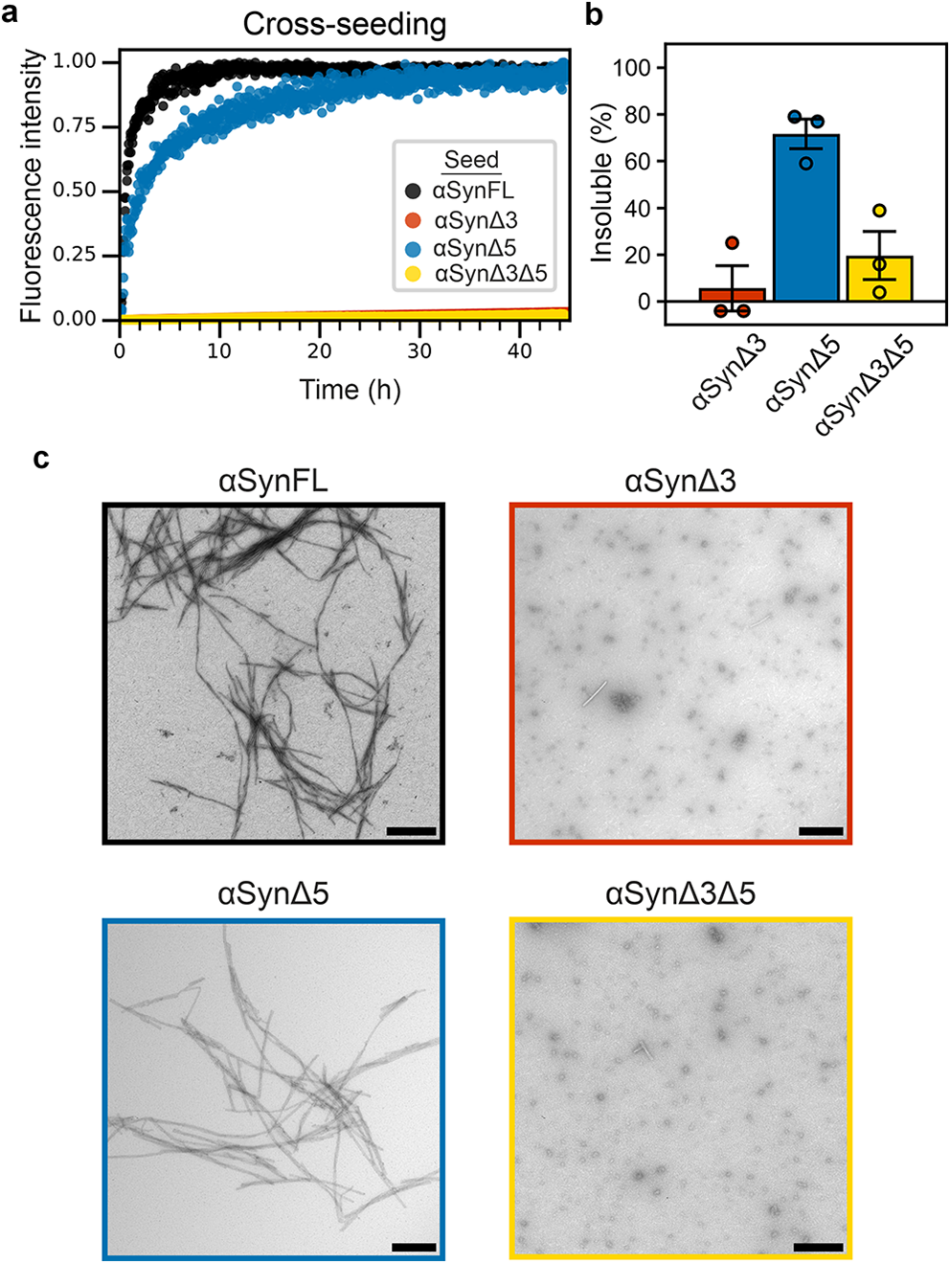
Cross-seeding of the alternative splice variants of αSyn with αSyn monomer. (a) Representative ThT fluorescence traces of αSynFL monomer and the fibril seed (type indicated in the key) in PBS. Data are normalized to the maximum intensity of the well if seeding occurred (based on ThT fluorescence, pelleting assay, and negative stain TEM) or to the maximum intensity measured from all conditions if seeding did not occur. (b) Quantification of the percent insoluble material formed during the cross-seeding reaction and (c) representative negative stain TEM images of the material formed. Scale bar, 250 nm.

These results show that the residues encoded by exon 5 have a stronger impact on the rate of *de novo* amyloid formation than those encoded by exon 3, but those encoded by exon 3 modulate recruitment of the αSynFL monomer to fibrils. Hence, the different regions of the αSyn sequence regulate different processes of amyloid formation, with the effect of removing one region being dependent on the context of the remaining sequence.

### The Ionic Strength Dependence of Amyloid Formation

Long-range interactions between the amphipathic N-terminal domain and highly acidic C-terminal domain of monomeric αSyn have been reported to regulate its amyloid formation.^52–55^ The splice variants have stark differences in their charge-related sequence properties (Table S7), suggesting that alternative splicing could affect the monomer conformation and, thus, the amyloidogenicity of αSyn. Specifically, there is a single His residue, one negatively charged and two positively charged residues in the 14-residue exon 3 (absent in αSynΔ3 and αSynΔ3Δ5), and ten negatively charged residues in the 28-residue exon 5 (absent in αSynΔ5 and αSynΔ3Δ5) (Figure 1c). To determine how the monomer conformation of the splice variants influences their amyloidogenicity, we next examined the effect of changing the ionic strength on the rate of the different pathways of amyloid formation for each splice variant.

The ionic strength dependence of amyloid formation of the different splice variants is shown in Figure 4a. All variants formed amyloid under all conditions, except for αSynΔ3 in 0 mM NaCl. Empirical fitting of the ThT fluorescence curves using a previously established general equation for accumulation of amyloid fibril mass (Figure S4, Experimental Section and eq 2)^64^ was used to extract the macroscopic rate parameters λ and κ (Figures 4b, S5, S6, and Table S8), which describe the collective rate of the primary and secondary pathways of amyloid formation, respectively. The primary pathway is the sequence of primary nucleation and elongation by which *de novo* amyloid fibrils first form, whereas the secondary pathway is the positive feedback cycle of secondary processes (secondary nucleation and fragmentation) and elongation that results in exponential accumulation of fibril mass in the growth phase.^64^ While the rate of the primary pathway (λ) decreases approximately 20-fold from 0 to 400 mM NaCl for αSynFL, there is little or no ionic strength dependence of λ for the other variants (Figure S5 and Table S8). By contrast, the rate of the secondary pathway (κ) of all variants increases at lower ionic strength for all variants and saturates at NaCl concentrations greater than 200 mM for all variants except for αSynΔ3 (Figure 4b). Our analyses of the fitting error (Figure S6) show us that we can be confident in the best-fit λ and κ values (Figures 4b and S5). Despite this, there is a high degree of inter-replicate variability in λ, likely arising from confounding well-to-well experimental variation (*e.g*., the surface of the beads used to promote primary nucleation).

**Figure 4:**
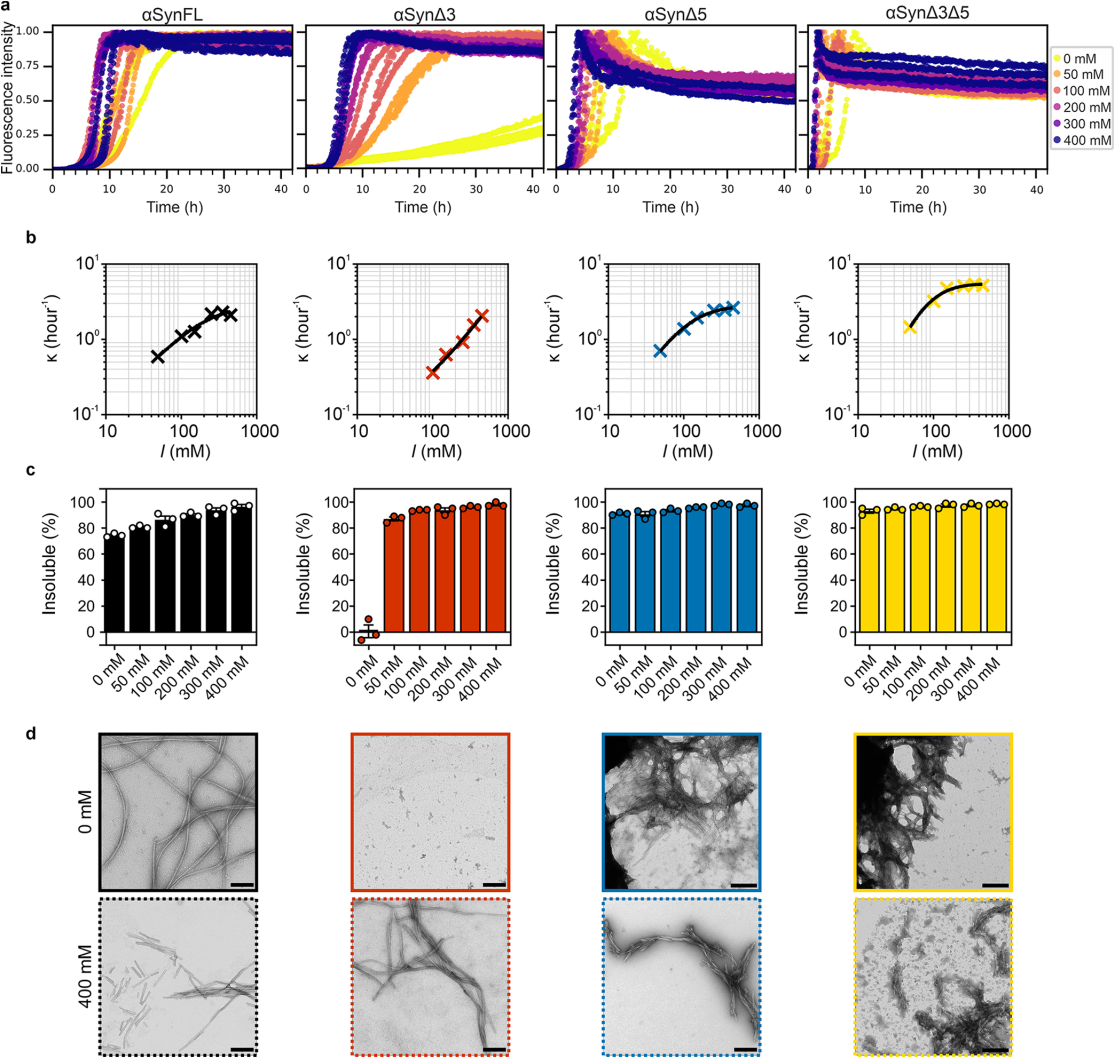
Ionic strength dependence of amyloid formation of αSyn and its splice variants. (a) Representative ThT fluorescence curves for the four splice variants (as indicated above each plot) in 20 mM sodium phosphate (pH 7.4) at NaCl concentrations ranging from 0 to 400 mM (shown in key). The starting αSyn monomer concentration was 100 μM. Data are normalized to the maximum intensity of the well, excluding αSynΔ3 at 0 mM NaCl, as a plateau was not reached within the 42 h of the experiment; in this case, data are normalized to the maximum intensity detected for the entire αSynΔ3 data set. (b) κ values derived from empirical fitting of the ThT curves using eq 2 (Experimental Section). Note that the error bars could not be plotted as they are smaller than the size of the symbol. The data are fitted by using eq 10 (Experimental Section) and shown as the solid lines on the plots. The plotted ionic strength (*I*) is that of the total buffer. (c) Quantification of insoluble material at the end of the incubation period for each splice variant at each ionic strength. Each data point is the result of one biological repeat, and the error bars are SEM. Note that in some cases the SEM is too small to be seen on the plots. (d) Representative negative stain TEM images of the material formed during the ThT assays in 0 or 400 mM NaCl. Scale bar, 250 nm.

The values of κ are notably different between the variants. While deletion of exon 3 can either increase or decrease κ depending on sequence context, deletion of exon 5 consistently causes an increase in κ, with a ∼1.2-fold increase in κ across all ionic strengths for αSynΔ5 compared with αSynFL, and a >2.5-fold increase in κ for αSynΔ3Δ5 compared with αSynΔ3. The difference between αSynΔ3Δ5 and αSynΔ3 is more extreme at lower ionic strengths, with κ 10-fold higher for αSynΔ3Δ5 in 50 mM NaCl. As expected from these data, the percent of insoluble material formed at the end of the ThT assay for αSynΔ3 is also dependent on ionic strength, with no fibrils forming in 0 mM NaCl, while fibrils resulted in 50 mM NaCl (Figure 4c,d, and Table S9). Negative stain TEM also showed that while αSynFL forms long amyloid fibrils in 0 mM NaCl, the fibrils are visibly shorter in 400 mM NaCl (although length could not be quantified due to fibril clumping), supporting the kinetic data that the rate of secondary processes increases with ionic strength (Figure 4d).

The discrepancy between the rate of fragmentation and the measured rate of secondary processes in *de novo* assembly can be calculated from the ratio between the observed and expected absolute change in the rate of elongation, *k*, i.e., (0.072–0.036)/(7.10–0.036) for αSynFL and (0.061–0.036)/(0.325–0.036) for αSynΔ3. This shows that the rate of fragmentation is approximately 200-times (αSynFL) and 10-times (αSynΔ3) too low to explain the κ values observed in *de novo* assembly (Figure S7), showing that secondary nucleation is the dominant process for αSynFL and αSynΔ3 under our conditions, consistent with previous observations.^65^

αSynΔ5 and αSynΔ3Δ5 flocculate during amyloid formation—shown by the decrease in ThT fluorescence signal in the plateau phase (Figure 4a) and negative stain TEM images (Figure 4d), consistent with previous observations with other C-terminal truncation variants.^9^ As a consequence, we were unable to perform the same analysis for these variants, leaving open the possibility that fragmentation could play a more important role in these sequences. However, as κ follows the same trend with ionic strength between the splice variants, it is most likely that the same mechanism controls their behavior. Hence, we propose that secondary nucleation, not fragmentation, is the specific secondary process that is governed by ionic strength.

To better understand the underlying mechanism of the influence of ionic strength on secondary nucleation in fibril formation, the ionic strength dependence of κ was fitted to two plausible mathematical models applying the Debye–Hückel theory to different steps of the fibril self-replication process (Experimental Section and Supporting Information). In the "Brønsted–Bjerrum"model, ions are assumed to stabilize monomer-fibril interactions, whereas in the “Free Energy Barrier” model, ions are assumed to stabilize a conformational transition state involved in templated conversion to the amyloid state. We note that these models do not mathematically distinguish between interactions/conversion on the fibril surface (i.e., secondary nucleation) or fibril ends (i.e., elongation), but as λ does not increase with ionic strength, we can conclude that the dominant change in interaction/conversion when interpreting these data is due to changes in the processes occurring at the fibril surface. Both models predict a positive relationship between κ and ionic strength, but only the “Free Energy Barrier” model was able to reproduce the observed saturation of κ at higher ionic strengths (Figures 4b, S8 and Table S10). This suggests that unfavorable electrostatic interactions, perhaps the alignment of like charges during fibril nucleation, contribute to the free energy barrier for templated conversion to the amyloid state (Δ*G* ^‡^ = Δ*G*_charged_^‡^ + Δ*G*_noncharged_^‡^), and screening of these unfavorable electrostatic interactions is responsible for the increase in κ with increasing ionic strength. The existence of a limit on κ at high ionic strength, on the other hand, intuitively suggests that there are also nonelectrostatic factors that limit the rate of secondary nucleation.

Using this model, we were also able to investigate the differences between the variants, separating out the electrostatic (Δ*G*_charged_^‡^) and nonelectrostatic (Δ*G*_noncharged_^‡^) contributions to the free energy barrier (Table S11). Most notably, the saturated (i.e., fully screened) value of κ is increased for αSynΔ3Δ5 relative to αSynFL and αSynΔ5 (Figure 4b). This suggests that the free energy barrier that remains when any unfavorable electrostatic interactions are screened out is lower for αSynΔ3Δ5 than for αSynFL or αSynΔ5, implying that exon 3 makes additional unfavorable contributions to the free energy barrier that are independent of the electrostatics. In agreement with this, fitting with the favored “Free Energy Barrier” model suggested that αSynΔ3Δ5 has a smaller Δ*G*_noncharged_^‡^ than αSynΔ5 (ΔΔ*G*_noncharged_^‡^ = −1.38 ± 0.16 RT). The lack of observed saturation up to κ = 2 h^–1^ for αSynΔ3 suggests that the same is true for this variant, and the model analysis also suggested that αSynΔ3 has a smaller Δ*G*_noncharged_^‡^ than αSynFL, although the margin of error is much larger due to uncertainty regarding the exact limit that κ tends to at saturation (ΔΔ*G*_noncharged_^‡^ = −4.52 ± 3.55 RT). Taken together, the results suggest that the residues encoded by exon 3 (^41^GSKTKEGVVHGVAT^54^) make an additional nonelectrostatic contribution to the free energy barrier for conversion from fibril-bound monomer to the amyloid state, although the molecular origins of this effect remain unresolved.

It is also striking that fibrils did not form for αSynΔ3 in 0 mM NaCl, whereas a quantitative (>87 ± 2%) conversion of monomer to insoluble material resulted at concentrations ≥50 mM NaCl (Figure 4c,d), and the κ values were globally smaller for this protein compared with the other variants at all ionic strengths (Figure 4b). The simplest explanation for this observation is that αSynΔ3 experiences additional inhibitory electrostatic interactions that either do not occur or are adequately compensated for in the other variants and are tunable by modifying the ionic strength. As αSynΔ3Δ5 also lacks the residues encoded by exon 3, yet does not display this behavior, it suggests that the reduction in κ for αSynΔ3 results from the presence of a complete C-terminal region (containing exon 5) in addition to truncation of the N-terminal region (by deletion of residues encoded by exon 3), which would maximize charge imbalance. This demonstrates that the consequence of exon splicing on the behavior of αSyn is dependent on the context of the remaining residues.

### The Ionic Strength Dependence of Monomer Conformational Properties

We next explored the influence of ionic strength on the conformational properties of the monomers of alternative splice variants of αSyn. Using flow-induced dispersion analysis (FIDA), we measured the average hydrodynamic radii (*R*_h_) of the αSyn splice variants at different ionic strengths (Figures 5a and S9). At the highest ionic strength (400 mM NaCl), at which concentration most electrostatic interactions are expected to be screened, all four variants had *R*_h_ values that were ∼0.3–0.4 nm smaller than predictions for fully unfolded proteins of the same lengths (Table S12),^66^ consistent with well-documented nonlocal interactions involving hydrophobic residues and/or transient secondary structure.^67,68^ However, at low ionic strength (0 mM NaCl), a further pronounced compaction was observed for αSynFL (consistent with previous investigations using paramagnetic relaxation enhancement NMR^17,52,55^ ) and for αSynΔ3, but not for αSynΔ5 and αSynΔ3Δ5 (Figure 5a and Table S12). For example, αSynFL compacted from a *R*_h_ of 3.28 ± 0.03 nm (400 mM NaCl) to 3.05 ± 0.02 nm (0 mM NaCl), whereas αSynΔ5 had corresponding *R*_h_ values of 2.91 ± 0.02 nm (400 mM NaCl) and 2.88 ± 0.01 nm (0 mM NaCl). This suggests that the residues encoded by exon 5 form nonlocal electrostatic interactions that drive global compaction of these variants at low ionic strength. This can be rationalized by the fact that ten negatively charged residues are encoded by exon 5. By contrast, inclusion or exclusion of the residues of exon 3 has little effect on the relationship between the ionic strength and *R*_h_.

**Figure 5:**
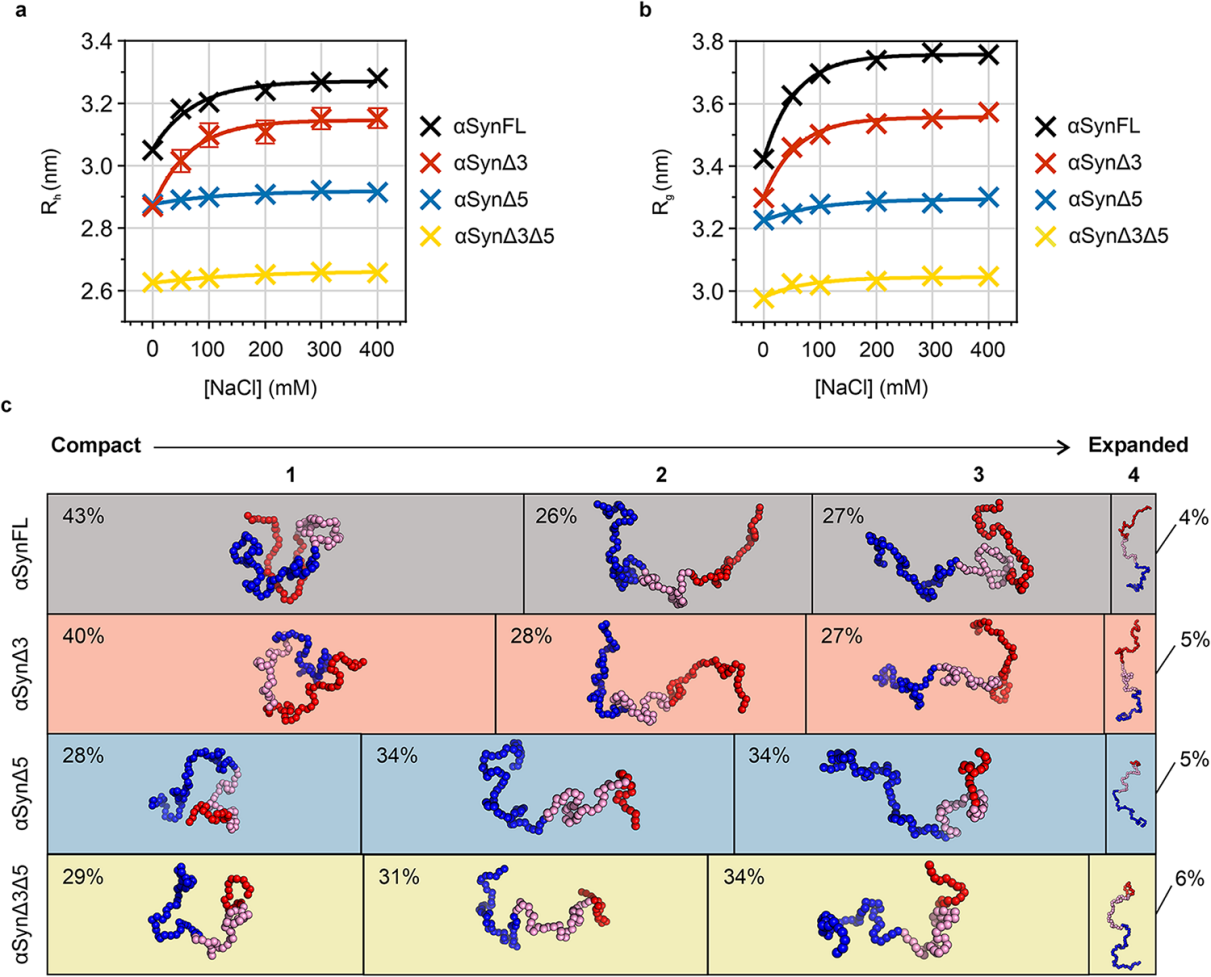
Monomeric conformers of the alternative splice variants of αSyn. (a) *R*_h_ values of the alternative splice variants of αSyn at different NaCl concentrations. Average *R*_h_ values for each condition were determined experimentally by FIDA (Experimental Section). Mean *R*_h_ values from at least eight Taylorgrams and the SEM are plotted. A one-phase exponential decay was fitted to the data in GraphPad Prism 10.1.2. (b) Average *R*_g_ values of alternative splice variants of αSyn at different ionic strengths in CALVADOS 2 simulations. Note that the ionic strength used for simulations is converted into equivalent NaCl concentration here, to aid comparison with panel a. (c) Classification of the conformational ensemble of the alternative splice variants of αSyn using spectral clustering, alongside representative conformers. The structural class 1 is the most compact cluster, with many interactions between the N-terminal, NAC, and C-terminal domains. Class 2 is characterized by a reduction in interactions between the C-terminal domain and the N-terminal/NAC region. Class 3 has fewer interactions between the N-terminal domain and the NAC/C-terminal region. Class 4 contains the most expanded conformations, which have the fewest interactions between all three domains. The percentage of the frames of each simulation of the alternative splice variants in each of the four structural classes is shown. The width of the boxes represents the proportion of frames that were categorized as being in the corresponding class, with percentages of frames indicated in the boxes. Data used here were from simulations at 49 mM ionic strength (equivalent to 20 mM sodium phosphate, 0 mM NaCl). The most representative frame (Experimental Section), in terms of global inter-residue distances, for each cluster for each variant is displayed within each box, with the N-terminal domain colored blue, the hydrophobic core domain (NAC) colored pink, and the C-terminal domain in red.

To characterize the conformational ensembles of the variants at different ionic strengths, and to better understand the link between sequence, monomer conformation, and the rate and mechanism of amyloid formation, we carried out coarse-grained molecular dynamics (MD) simulations using the CALVADOS 2 force field.^57,58^ The CALVADOS 2 simulations predicted a change in compaction with sequence and ionic strength that shows a striking resemblance to the experimental FIDA data (Figure 5b and Table S13). In the simulation results, we characterized compaction using the radius of gyration (*R*_g_) rather than *R*_h_, as the former can be exactly calculated from analysis of simulation trajectories, whereas postsimulation analysis of *R*_h_ remains challenging^69^ (however, *R*_g_ and *R*_h_ are generally expected to scale closely with one another^70,71^ ). Analysis of the Flory exponents (ν), which provide a length-independent measure of chain compaction,^72^ also confirmed a compact state (ν < 0.5) for αSynFL and αSynΔ3 at low ionic strength, but an intermediate degree of compaction (ν = 0.54) for αSynΔ5 and αSynΔ3Δ5 (Table S14).

We next analyzed the simulation trajectories to obtain intramolecular C_α_-C_α_ contact probability maps, showing the proportion of time that each pair of residues spends within a threshold distance (20 Å) of each other. In agreement with previous experimental studies,^17,52,55,73^ the contact maps of αSynFL and αSynΔ3 had strong contact probabilities between the N-terminal and C-terminal domains at low ionic strength (Figure S10). These interactions were abolished at higher ionic strengths (Figure S11) and were attenuated at all ionic strengths for the αSynΔ5 and αSynΔ3Δ5 variants, which lack exon 5 and thus 28 of the 45 residues of the C-terminal domain (Figure S10). In addition, αSynΔ3 had a mild attenuation of interactions between the C-terminal domain and residues ∼20 to 40 that juxtapose the missing exon 3, although the overall effect on the N- to C-terminal domain interaction propensity was much smaller than that caused by deletion of exon 5 (Figure S10). Thus, the simulations show that favorable electrostatic interactions between the amphipathic N-terminal domain and acidic C-terminal domain, which contains exon 5, drive compaction of αSynFL and αSynΔ3 at low ionic strength.

To explore in more detail how the conformational ensembles differ between splice variants, we used spectral clustering to classify conformers within the simulation trajectories based on the similarity of their C_α_–C_α_ distances (Experimental Section). This allowed us to identify distinct compact, partially compact, and expanded species within the conformational ensembles of the αSyn variants (Figures S12 and S13). It is important to note that αSyn has a relatively smooth conformational energy landscape in our simulations, so that these states represent subdivisions of a spectrum of conformations in the energy landscape rather than well-separated energy basins. Across all variants, we reproducibly identified four structural classes: a compact class involving N–C interactions; two partially compact classes with expanded N- or C-terminal domains; and an expanded class (Figure 5c). By quantifying the distribution of conformers (i.e., simulation frames) across each of these structural classes, we identified that at 49 mM ionic strength (equivalent to 20 mM sodium phosphate buffer, pH 7.4, 0 mM NaCl) αSynFL and αSynΔ3 were in their most compact class in 43 and 40% of the frames, respectively, while αSynΔ5 and αSynΔ3Δ5 were only in their most compact class for 28 and 29% of the frames, respectively (Figures 5c and S13). This suggests that the presence of exon 5 at low ionic strength skews the conformational distribution of αSyn toward an enhanced population of structurally interrelated compact species involving long-range N–C interactions.

Overall, the results of the coarse-grained MD simulations suggest that a more expanded conformational ensemble with fewer long-range N–C interactions correlates with a higher rate of secondary nucleation and more rapid amyloid formation.

### Monomer Conformation Correlates with Secondary Nucleation of αSyn Amyloid Formation

We next explored whether and how the conformational properties of the different αSyn monomers correlate with changes in their amyloid-forming ability at different ionic strengths. We examined the correlation between the experimental self-assembly data and three measures of the compaction and shape of the different αSyn variants in the CALVADOS 2 simulations: the Flory exponent (ν), asphericity (Δ), and prolateness (S) (Table S14). We focused on these metrics, as they are independent of chain length. Examples of conformations with different Δ and S are shown in Figure S14.

We calculated these metrics for the different alternative splice variants across all ionic strengths tested and performed a Spearman’s rank analysis with the parameters extracted from the amyloid formation assays: rate of the primary pathway (λ), rate of the secondary pathway (κ), and the percentage of insoluble material at the end of the experiment (Figures 6 and S15). The results were striking, revealing a clear and strong correlation between monomer conformation and amyloid formation. Although this correlation was evident between all three of the conformational properties (Δ, S, and ν) and both κ and percentage of insoluble material, the strongest correlations were identified between the prolateness of the monomer (S) and secondary pathway of amyloid formation (κ) and also between the percentage of insoluble material and ν, which each have a correlation coefficient of 0.85. Interestingly, the primary pathway of amyloid formation (λ) correlated more weakly with Δ, S, and ν (correlation coefficients of 0.60, 0.68, and 0.32, respectively). Furthermore, for the latter correlations with λ, we note that when we consider individual variants, rather than the combined data, the correlations appear to disappear or even reverse (Figure S15), an example of Simpson’s paradox.^74^ Importantly, the positive correlations between κ and the conformational properties of the monomer are consistent both within and upon combining data for the different variants, so we can be more confident in interpreting these findings. Overall, this analysis suggests that the global link between monomer conformational properties and amyloidogenicity is specific for the secondary pathway of amyloid formation (specifically relating to the effect on secondary nucleation) and that any relationship between ionic strength and the rate of the primary pathway is not shared among the four variants tested here.

**Figure 6:**
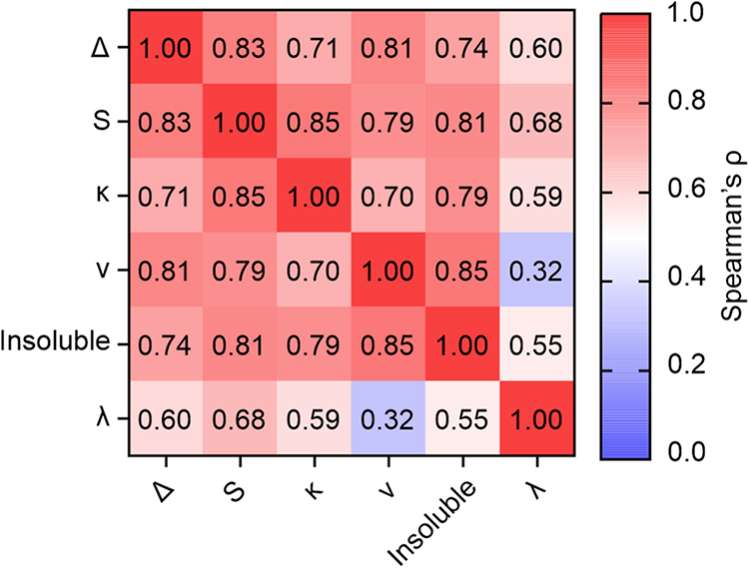
Spearman’s ρ correlation coefficient heatmap of the parameters extracted from the ThT assay (λ, κ, and insoluble material) and those predicted based on the CALVADOS 2 simulations (Δ, S, and ν).

The calculated κ and predicted S values for αSynΔ5 and αSynΔ3Δ5 are larger than those for αSynFL and αSynΔ3 at all ionic strengths tested, which supports the notion that the C-terminal domain of αSyn protects against amyloid formation, perhaps by driving compaction and shielding the hydrophobic NAC domain.^8^ It should be noted, however, that the results from this analysis do not explain the differences between αSynFL and αSynΔ3, as the fitted κ values indicate that secondary nucleation is consistently faster for αSynFL compared with αSynΔ3 at all ionic strengths tested, yet αSynΔ3 is predicted by CALVADOS 2 to be relatively more expanded than αSynFL across the same range of ionic strengths. This suggests that the residues encoded by exon 3 (41 and 54) may play a role in regulating the rate of amyloid formation that is distinct from changes to the overall expansion of the protein, perhaps due to differences in the regions that form interactions more frequently (Figure S10).

Finally, we performed a Spearman’s rank analysis to explore how inter-residue distances between all residue pairs correlate with the rate of the secondary processes, κ, for each variant across all ionic strengths (Figure S16). To determine if these relationships are conserved between the splice variants, we performed this analysis on data relating to inter-residue pairs common to all four variants (residues 1–40, 55–102, and 131–140 – yielding 1953 unique residue pairs) at all six ionic strengths. This revealed distinct correlation patterns for different regions of αSyn (Figure 7a). Of note, increased inter-residue distances within the hydrophobic NAC domain correlate positively with κ, supporting the notion that increasing solvent-exposure of NAC is associated with an increased rate of amyloid formation via secondary nucleation. The residue pair whose C_α_-C_α_ distance correlates most positively with κ is Q79-E83 (Figure 7b), although nearby residue pairs are also strongly correlated with κ. By contrast, the inter-residue distances within the C-terminal domain correlate negatively with κ (i.e., closer distances are associated with an increase in κ). A more complex picture is seen in the N-terminal domain, with a mixture of local compaction and expansion correlating with κ (Figure 7a), likely due to differences between the individual variants in this region (Figure S16). The residue pair distance that correlates most negatively with κ is Y133-A140 (Figure 7c), although the effect was broadly distributed across all C-terminal residues that were included in the analysis and thus likely encompasses the acidic C-terminal domain as a whole. Together, this analysis demonstrates that local expansion of NAC (particularly between Q79-E83) and compaction of the C-terminal domain correlate with a high rate of the secondary pathway of amyloid formation, and *vice versa*, enabling us to visualize the conformations of monomers associated with either a low (Figure 7d) or high (Figure 7e) value of κ and hence more rapid fibril formation via secondary nucleation.

**Figure 7:**
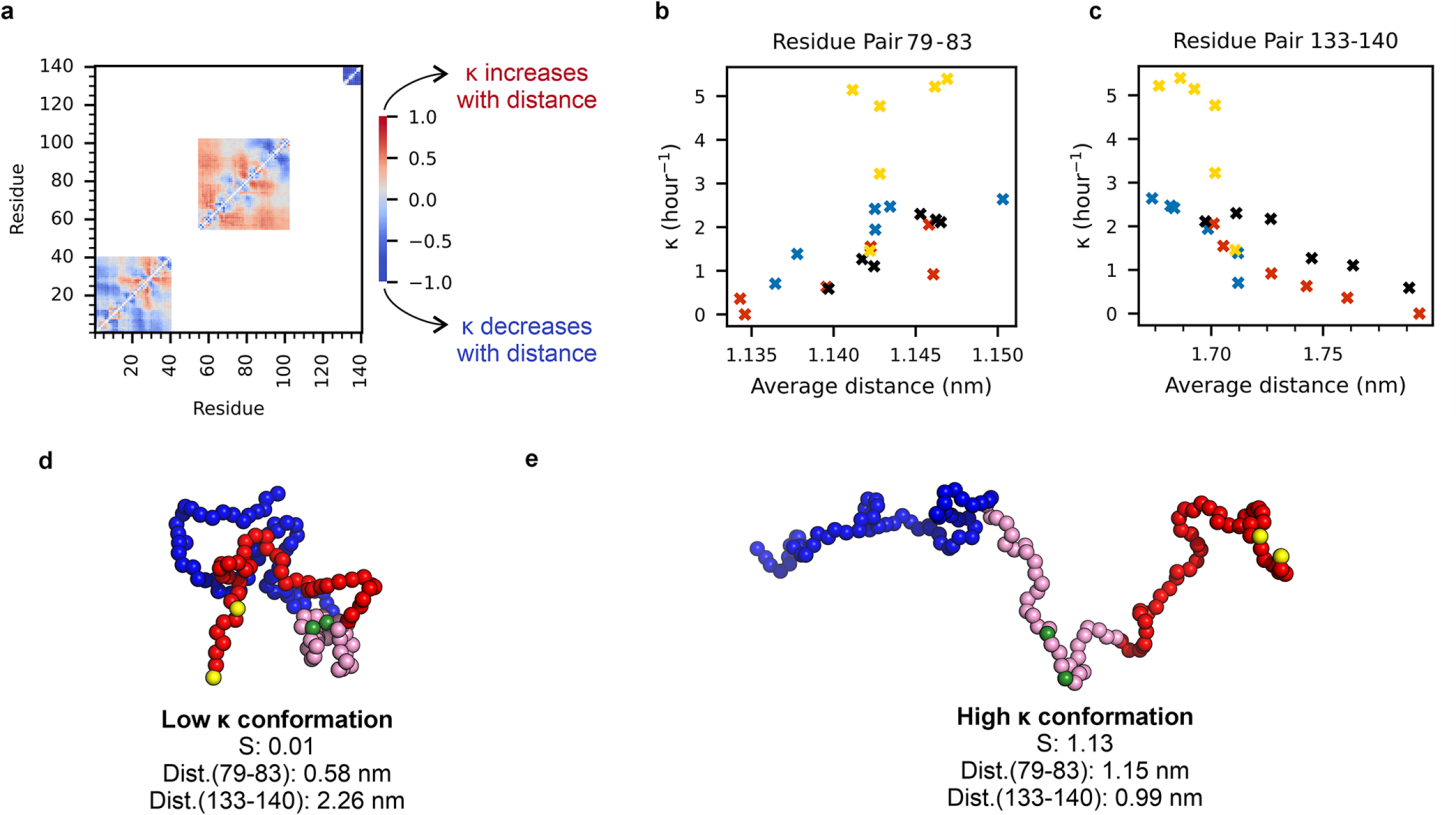
Inter-residue distances correlate with secondary nucleation in amyloid formation. (a) Spearman’s rank heatmap of the inter-residue distances between all residue pairs of the alternative splice variants of αSyn from CALVADOS 2 simulations with experimentally derived κ values. Residues encoded by exons 3 or 5 and residue pairs that cross potentially spliced-out regions are masked to prevent confounding results. (b) κ versus the average distances derived from the CALVADOS 2 simulations for the residue pair Q79-E83, whose inter-residue distance correlates the most positively with κ. (c) As in (b) for residue pair Y133-A140, the residue pair that correlates the most negatively with κ. (d) Representative low κ state conformation of αSynFL, with a low S, short distance between residues 79–83, and long distance between residues 133–140. (e) Representative high κ state conformation of αSynFL, with a high S, a long distance between residues 79–83, and a short distance between residues 133–140. In panels d and e, the N-terminal domain is shown in blue, the hydrophobic core domain is shown in pink, and the C-terminal domain is shown in red. Residues 79 and 83 are shown in green, and residues 133 and 140 are shown in yellow.

## Discussion

### Alternative Sequences, Conformational Ensembles, and Rates of Amyloid Formation

Here, using CALVADOS 2 simulations,^57,58^ we provide a robust link between the global conformation of monomeric αSyn (as judged by the parameters: ν, Δ, and S) and the rate of the secondary nucleation of amyloid formation that is displayed by all alternative splice variants analyzed here (Figure 8). The compaction of αSyn monomers at low ionic strength (Figure 8a) has been shown previously using paramagnetic relaxation enhancement NMR experiments,^17,52,55^ and has been suggested to shield the NAC region and inhibit secondary nucleation.^75^ In fact, αSyn­(K6A;K10A;K12A), which attenuates electrostatic interactions between the N- and C-terminal domains, has been reported to be more expanded than αSynWT and forms amyloid at a faster rate.^75^ Furthermore, we note that the notion of monomer conformation being an important determinant of amyloid formation has been described previously for other amyloidogenic proteins, including tau whose conformational expansion is also associated with the acceleration of amyloid formation,^76^ while compaction of the polypeptide chain resulting from Zn^2+^ binding (measured using ion mobility mass spectrometry) leads to an enhanced rate of amyloid formation for αSyn.^77^

**Figure 8:**
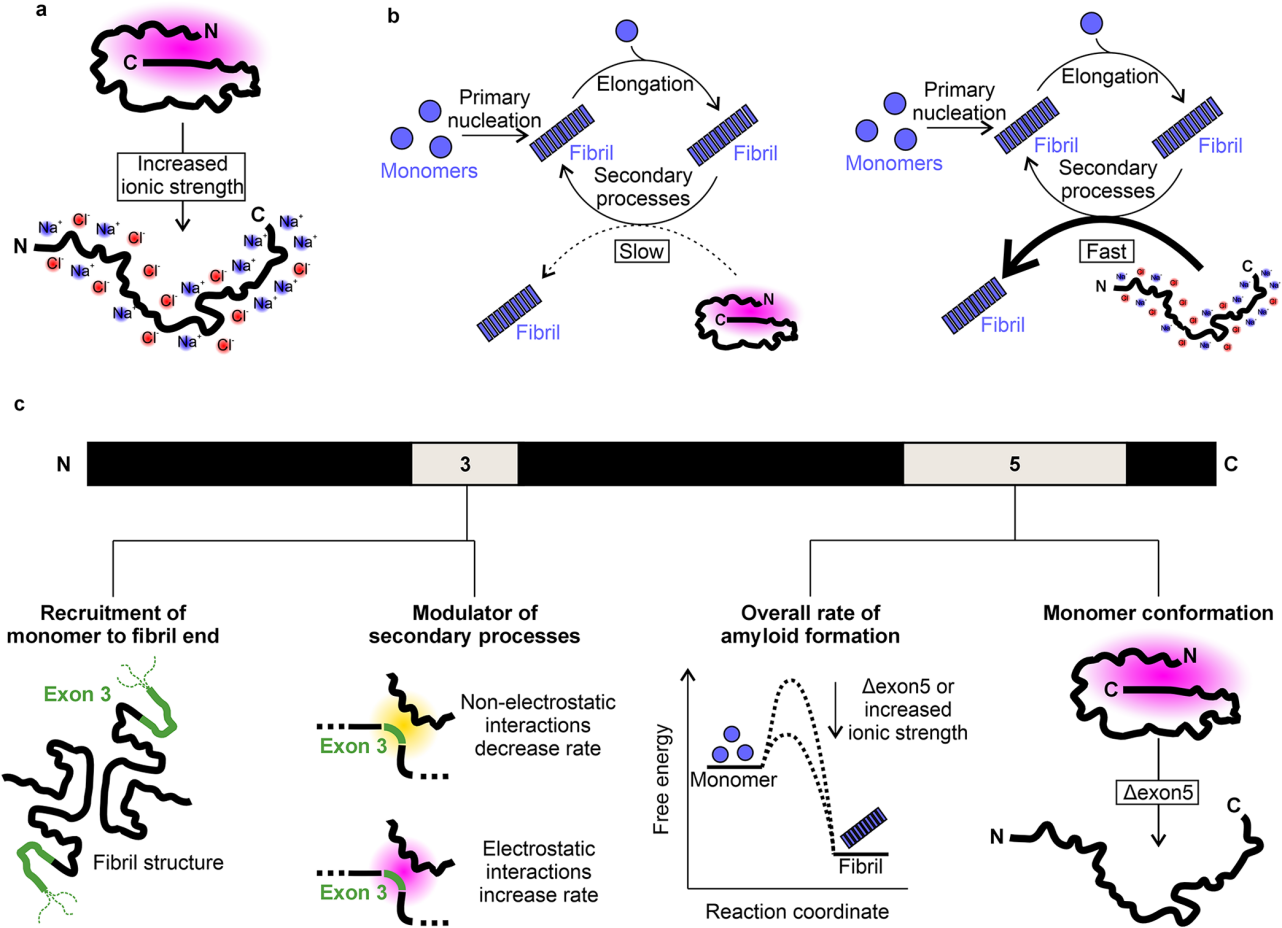
Importance of monomer conformation in amyloid formation of the alternative splice variants of αSyn. (a) Under low ionic strength conditions, electrostatic interactions, particularly between the N- and C-terminal domains of αSyn, cause compaction of the monomer. As ionic strength increases, these electrostatic interactions are screened, facilitating monomer expansion. (b) Amyloid formation of αSyn is achieved via primary nucleation from monomers, fibril elongation, and secondary nucleation. The more expanded monomers undergo secondary nucleation faster. (c) Summary of the findings on the roles of exons 3 and 5 in modulating the conformational ensembles of monomeric αSyn and the effects on amyloid formation kinetics. Exon 3 is required in the fibril for recruitment of αSynFL monomers (Figure 3) and modulates the rate of secondary nucleation (Figure 4). Exon 5 regulates the overall rate of amyloid formation by protecting the NAC domain (Figure 2) and altering the monomer shape and compaction (Figure 5).

Our analyses support the model^52^ that global compaction is driven largely by interactions between the N- and C-terminal regions of αSyn, and demonstrate that a more expanded monomer (and one with higher prolateness) correlates with increased κ (Figure 8b). The strong correlation of prolateness with secondary nucleation is interesting, as prolate conformers are “stretched out” and thus have an exposed NAC region (Figures 7e and S14b). On the thermodynamic side, it is also interesting that the Flory exponent (ν) correlates most strongly with the percent insoluble material (Figures 6 and S15), as polymer theory predicts that ν depends on the affinity of the polypeptide chain for itself rather than the solvent, and is thus closely related to solubility.^72^ That the increased prolateness associated with an increase in the ionic strength does not also cause an acceleration of primary nucleation, as indicated by λ, is notable. For αSynΔ3, αSynΔ5, and αSynΔ3Δ5, we observe minimal changes in λ over the range of ionic strengths tested here, suggesting that an exposed NAC region is not needed to form the initial interactions involved in primary nucleation and that electrostatics do not strongly affect the energy barriers for primary nucleation and elongation, at least under our conditions. The one exception to this is αSynFL, where there appears to be a weak inverse correlation between the ionic strength and the rate of primary nucleation. This could reflect screening out of favorable interchain electrostatic interactions between the N- and C-termini of different monomers, which enable primary nucleation to occur.

We propose that the kinetic effects of changing ionic strength on amyloid formation of all four variants observed here occur via a mechanism in which conformational changes in the monomer and/or fibril fuzzy coat result in more amyloid-compatible conformations at higher ionic strength. The influence of ionic strength on the rates of amyloid formation by αSyn has been investigated previously,^9,78–83^ and some studies have observed a reduction in secondary processes with increasing ionic strength,^78^ or a low rate of secondary processes relative to primary nucleation,^61^ in contrast to the observations presented here. Differences in pH^79^ (which affects the rates of both elongation and secondary nucleation^61^), or whether the experiments were shaking or quiescent (affecting fragmentation and secondary nucleation rates), could rationalize these differences. While an increase in the rate of the secondary pathway (κ) is observed at increased ionic strength, it should also be noted that this is accompanied by a decrease in the rate of the primary pathway (λ) for αSynFL (Figure S5), which likely explains the discrepancies with previous reports. Our observation that expansion of αSyn correlates with accelerated amyloid formation, although supported by other work,^52^ also contrasts with previous reports that have shown (using Zn^2+^ binding and ion mobility mass spectrometry (IM-MS)) that compaction of αSyn by binding of metal ions is coupled with faster kinetics of amyloid formation;^77,84^ suggesting that metal ion binding, sequence variants, and increasing NaCl concentrations differently affect the processes of amyloid formation. Hence, the nature of the local and global compaction appears to be crucial in determining the amyloid potential and pathways of αSyn aggregation. As such, the effect of the ionic strength on the amyloid kinetics of αSyn is highly context dependent.

We note that additional underlying factors may also contribute to the connection between the ionic strength and amyloid formation kinetics. Indeed, changes in the ionic strength could also alter the properties of the fibril surface that catalyzes the conversion of monomer to amyloid via secondary nucleation. Some of the N-terminal region and most of the C-terminal domain of αSyn are disordered in αSyn amyloid fibril structures^62^, and, given the high proportion of charged residues in these domains (Figure 1c), their conformational properties will likely also be altered by changes in ionic strength. Similarly, the influence of the ionic strength on the colloidal properties of fibrils of the alternative splice variants of αSyn remains unknown, and it is possible that changes in charge screening may alter the propensity for fibril–fibril interactions. Hence, changes in the fuzzy coat in response to ionic strength changes may contribute to the observed effects on the rate of secondary nucleation by changing the catalytic site and/or by reducing the number of accessible sites on the fibril surface.

Our analysis using CALVADOS 2 also identified two key regions in the αSyn sequence in which inter-residue distances strongly correlate with κ, suggesting that the local conformation of these regions affects the rate of secondary nucleation. Specifically, an expanded NAC and compact C-terminal domain are correlated with a high κ and *vice versa*. This accords with proposals that exposure of the NAC region facilitates the transition to an amyloid state,^52^ compaction of the residues at the C-terminus of αSyn (*e.g*., at lower pH) is associated with an increase in amyloidogenicity,^53,85^ and that the C-terminal domain exerts a modulating effect on NAC.^8,9^ Since our data show that these changes specifically correlate with the rate of the *secondary* pathway (i.e., secondary nucleation and elongation), it appears that this enhanced activity lies in the effects on the structural conversion of monomers by fibrils, rather than primary nucleation itself. Although there are several residue pairs in NAC and the C-terminal domain that correlate strongly with κ, it is striking that the inter-residue distance that correlates most strongly with κ is Q79-E83, given the identification of the E83Q variant in a patient with DLB.^86,87^ Similarly, the importance of the Glu at position 83 in amyloid formation kinetics was also recently demonstrated.^88^ Biophysical characterization has revealed not only that the E83Q substitution causes a shift to more extended monomer conformations (as measured by IM-MS), but interactions specifically with Q79 are altered in this variant (as monitored by NMR chemical shift perturbations).^87^ Furthermore, the E83Q variant forms amyloid more rapidly than does αSynWT, and amyloid fibrils of this disease variant are substantially shorter than those of αSynWT, consistent with an enhanced rate of secondary nucleation. Together the characterization of the E83Q variant supports our proposal that the Q79-E83 residue pair, as well as other aspects of local conformation in the NAC and C-terminal regions, are important determinants of the rate of the secondary nucleation in amyloid formation.

### Roles of Residues Encoded by Exons 3 and 5 in Amyloid Formation of αSyn

The results presented support previous studies that have shown that deletion of residues encoded by exon 5 accelerates amyloid formation of αSyn,^47,49^ consistent with literature precedents that the C-terminal domain of αSyn monomers can protect the hydrophobic core domain from amyloid formation.^8^ Our results also show that residues in exon 3 (^41^GSKTKEGVVHGVAT^54^) in the fibril are required for the recruitment of αSynFL monomers in seeded fibril growth reactions, consistent with recently reported findings.^49^ The finding that αSynΔ5 is the only splice variant capable of cross-seeding αSynFL monomers is surprising, given the proposed model that the N-terminal 11 amino acids of the monomer bind to the C-terminal domain in the fibril ‘fuzzy coat’,^75,89^ of which 28 residues are missing in the αSynΔ5 variant (Figure 1). It has been noted previously that seeds with C-terminal truncations can indeed recruit αSynFL monomers, albeit more slowly than self-seeding.^9^ Instead, we find that it is residues 41–54 of the fibril that need to be present to facilitate efficient seeding of αSyn monomers (Figure 3). Notably, these 14 residues are present in the fibril core in many of the >140 αSyn amyloid fibril structures,^62,63^ which suggests why αSynFL is unable to adopt the fold of αSynΔ3 amyloid via elongation. Furthermore, we note that proteinase K digestion of the fibrils of αSynFL and αSynΔ5 suggests that they contain the same residues in the fibril core.^49^ Although we do not have information on the fibril core structures of the alternative splice variants generated in this work, residues 103–130 are not resolved in any of the published amyloid fibril structures,^62,63^ this ultimately allows us to rationalize why the deletion of these residues does not affect the ability of αSynFL to adopt the fold of αSynΔ5 amyloid fibrils in the process of elongation. Additionally, the involvement of the N-terminal residues of the fibril in seeded growth has been observed previously,^10^ with truncated proteins αSyn36–140 and αSyn41–140 unable to recruit αSynFL monomers. The implications of these findings in terms of PD pathogenesis are that if αSynΔ5 is indeed capable of forming amyloid in the brain, it may also have the capacity to recruit the more abundant αSynFL, triggering the chain reaction of amyloid formation and subsequent cell-to-cell spreading of amyloid in disease.

## Conclusions

We have demonstrated that the higher propensity of αSynΔ5 and αSynΔ3Δ5 to form amyloid *in vitro* can be rationalized from differences in monomer conformations compared with αSynFL, particularly global conformation in terms of prolateness, in addition to local conformation in the NAC and C-terminal regions and the extent to which the NAC is shielded by the rest of the protein sequence. Although the physiological and pathological importance of the αSyn splice variants remains unknown, our findings suggest that the splice variants could be involved in disease pathogenesis, particularly αSynΔ5, which we have shown can recruit αSynFL monomers via fibril elongation and self-propagate most rapidly via secondary nucleation. If these variants do indeed prove to be involved in the pathogenesis of synucleinopathies, they might be targeted for disease treatments; for example, with RNA interference technology, which would be designed to target specific toxic isoforms of *SNCA* and facilitate cleavage of the relevant mRNA to prevent it from being translated.^90^ This technology has already proved successful in reducing levels of another amyloidogenic protein, transthyretin, in familial amyloid polyneuropathy.^91^

From an evolutionary perspective, the sequence patterning that facilitates the promiscuous functions of αSyn^35^ has enabled the N- and C-terminal domains to mitigate aggregation driven by the NAC domain. Our results suggest that monomeric αSyn must exist in a carefully balanced equilibrium of conformations, where changes in sequence or modulation of the environmental conditions, probed here by changes in ionic strength, can critically determine its amyloidogenicity.

## Experimental Section

### Generation of Plasmids

DNA plasmids designed for recombinant protein expression of the alternative splice variants were generated from the pET23a vector encoding αSyn (gifted by Professor Jean Baum, Department of Chemistry and Chemical Biology, Rutgers University, NJ). To generate the αSynΔ3 variant, primers were designed to delete amino acid residues ^41^GSKTKEGVVHGVAT^54^ by Q5 site-directed mutagenesis (NEB). Similarly, for the generation of αSynΔ5, the primers were designed for the deletion of residues ^103^NEEGAPQEGILEDMPVDPDNEAYEMPSE^130^ from the αSynFL construct. To generate αSynΔ3Δ5, these latter primers were used on the αSynΔ3 construct. Q5 site-directed mutagenesis was carried out using the primers (Table S15), subsequently followed by treatment with kinase, ligase, and DpnI resulting in DNA circularization and template removal. Validation of the correct deletion was achieved by transforming *Escherichia coli* DH5α cells, plasmid purification using a Miniprep kit (Qiagen), followed byDNA sequencing (Source Bioscience).

### Protein Expression and Purification

The plasmids generated above were used for the expression of recombinant proteins of all variants of αSyn. *E. coli* BL21 DE3 cells were transformed with the plasmid of interest by heat-shock at 42 °C, and bacteria were grown on LB-agar plates containing carbenicillin (100 μg/mL) overnight at 37 °C. The following day, 100 mL of carbenicillin-containing LB medium was inoculated with a single colony and incubated for ∼16 h (overnight) at 37 °C, 200 rpm. The next day, 15 mL starter culture was added per 1 L of LB medium (containing 100 μg/mL carbenicillin) and placed in an incubator (shaking at 200 rpm at 37 °C). Once the OD_600_ reached ∼0.6, 1 mM isopropyl-β-d-thio-galactopyranoside was added to induce expression of the αSyn protein. The cultures were placed back in the shaking incubators and left to express protein for 4–5 h. After this time, the culture was centrifuged at 5000 rpm (rotor JA 8.1) at 4 °C, and the cell pellet was stored at −20 °C until further use.

The cell pellets were thawed and homogenized in lysis buffer (20 mM Tris-HCl, pH 8.0, 2 mM MgCl_2_, 5 mM DTT, 1 mM PMSF, 2 mM benzamidine, 100 μg/mL lysozyme, and 20 μg/mL DNase) and incubated on a roller at room temperature for 30 min. After this time, the homogenate was boiled at 80 °C for 10 min and subsequently centrifuged at 35,000*g* for 30 min. 30% (*w/v*) ammonium sulfate was then added to the resulting supernatant fraction, and the sample was incubated on a roller at 4 °C to facilitate protein precipitation. The sample was then centrifuged again at 35,000*g* for 30 min at 4 °C, with the pelleted fraction retained afterward. The precipitation and centrifugation steps were repeated once more, and the resulting pellet was stored at −20 °C until further processing.

The sample was purified by anion exchange chromatography. The pellet was thawed and resuspended in buffer A: 20 mM Tris-HCl (pH 8.0 for αSynFL and αSynΔ3, and pH 9.0 for αSynΔ5 and αSynΔ3Δ5 due to differences in the isoelectric points of the proteins). Anion exchange chromatography was carried out using a Q-Sepharose column packed in-house. Protein was applied to the column and washed with two column volumes (CV) of 20 mM Tris-HCl at the relevant pH. A gradient with buffer B (20 mM Tris-HCl, 1 M NaCl at pH 8.0 or 9.0 as above) was then applied to the column up to 50% (*v/*v) final concentration over two CVs. The same ratio of buffers A and B was applied to the column for an additional two CVs, before washing the column with 100% buffer B over two CVs. The eluted αSyn proteins were then dialyzed into 5 mM ammonium bicarbonate, lyophilized, and stored at −20 °C until further use.

The protein was further purified using size exclusion chromatography on a HighLoad26–60 Superdex 75 prep grade gel filtration column. The lyophilized protein was first dissolved in phosphate-buffered saline (137 mM NaCl, 2.7 mM KCl, 8.1 mM Na_2_HPO_4_, and 1.5 mM KH_2_PO_4_, pH 7.4) at a concentration of 2 mg/mL and injected onto the column in 5 mL loading volumes using a 50 mL Superloop. Collected protein was dialyzed into 5 mM ammonium bicarbonate, lyophilized, and stored as described above. Correct and pure protein was confirmed using SDS PAGE and ESI-mass spectrometry.

### Thioflavin T Assays

Lyophilized protein was dissolved in the buffers defined in the figure legends and centrifuged at 16,602*g* for 30 min at 4 °C to remove insoluble material. Protein concentration was determined by measuring the A_280_ using an extinction coefficient (ε) of 5960 M^–1^ cm^–1^ for αSynFL and αSynΔ3, and ε of 4470 M^–1^ cm^–1^ for αSynΔ5 and αSynΔ3Δ5. For the *de novo* ThT assays, the protein at the concentration defined in the figure key was mixed with 20 μM ThT, and 100 μL was added to the assay plate (Corning, 3651) in triplicate. A single 3 mm Teflon polyball (PolySciences) was added to each well of the assay plate, which was subsequently placed in the FLUOstar Omega Plate Reader (BMG Labtech). The ThT assay was carried out at 37 °C for 45 h with orbital shaking (600 rpm). The fluorescence of each well of the plate was measured using an excitation wavelength of 444 nm with the emission monitored at 480 nm.

For the seeding ThT assays, the starting monomer concentration was 50 μM. The fibril seeds were prepared by taking the fibrils generated in the *de novo* ThT assay and subjecting them to two rounds of 30 s sonication using a Cole-Parmer-Ultraprocessor sonicator and 40% power. The fibril seeds were added to the seeding experiments at a concentration of 5 μM (monomer equivalent). ThT assay was performed as above, in the absence of the Teflon polyball and with no shaking in the FLUOstar OPTIMA Plate Reader (BMG Labtech).

The *T*_50_ values for the ThT assays carried out at different monomer starting concentrations are defined as the first data point that crosses the threshold of 50% of the maximum ThT signal of the normalized curve. Values for the scaling exponent (Table S3) were determined by performing nonlinear regression of the *T*_50_ values versus the starting concentration of αSyn using the equation

1 $$y=ax^{\gamma }$$

where *a* and γ are the scaling coefficient and exponent, respectively. Fitting and calculation of the 95% confidence intervals (CI) were carried out in GraphPad Prism 10.3.1.

For measurements of the ionic strength dependence of amyloid formation using ThT assays, the starting monomer protein concentration was 100 μM. The ThT assays were carried out as described above for the *de novo* experiments.

ThT data from the ionic strength dependence experiment were fitted to the equation^64^

2 $$y=1-{\left[1+\frac{\lambda^{2}}{2\kappa^{2}\theta }e^{\kappa t}\right]}^{-\theta }$$

where *y* = normalized ThT intensity, *t* = time, and λ, κ, and θ are fitted parameters with 0 ≤ θ ≤ 3. λ and κ were fitted for each replicate, but θ was fitted globally for each variant. Note that only data prior to the maximum fluorescence value was used for fitting; this is to account for the decreases in signal observed during the plateau, particularly for αSynΔ5 and αSynΔ3Δ5, which is potentially due to flocculation and is not accounted for in current models of amyloid formation.

To test whether fragmentation is the dominant secondary process in our experiments, we first performed a ThT assay using 100 μM monomer in 20 mM sodium phosphate and 100 mM NaCl, pH 7.4, at 37 °C under orbital shaking at 600 rpm in the presence of a Teflon bead. At 30 h, we collected the fibrils and added these (without sonicating) to fresh monomer of the same variant (50 μM monomer plus 10 μM monomer equivalent concentration of seed). The kinetics of self-seeding was assessed as described for the seeding assays described above. 15 h later (45 h after the start of the first *de novo* ThT assay), we collected fibrils from another well and tested the self-seeding potential again.

If fragmentation is the dominant secondary process in *de novo* fibril self-assembly, then there is a precise expected relationship between κ and the rate of change of the seeding potency of a plateau-phase fibril sample due to fragmentation. In elongation-dominated seeding, monomer disappears exponentially at a rate dependent on the seed fibril number concentration *P*_seed_ and elongation rate constant *k*_+_.^92^

3a $$m\left(t\right)=m_{0}e^{-\mathrm{kt}}$$

3b $$k=2k_{+}P_{\mathrm{seed}}$$

where *m*(*t*) is the monomer concentration as a function of time, and *m*_0_ is the initial monomer concentration in the seeded assay. This rate of disappearance can be influenced by incubating the fibrils longer before use, allowing them to fragment more. After a time delay Δ*t*, the concentration of fibril ends will have increased by an amount proportional to the fragmentation rate *k*_frag_ and seed fibril mass *M*_seed_.^92^

4 $$P_{\mathrm{seed}}^{\prime }=P_{\mathrm{seed}}+k_{\mathrm{frag}}M_{\mathrm{seed}}\Delta t$$

This means that if fragmentation occurs at a significant rate, a fibril sample that has been incubated for longer before use will have more fibril ends and thus greater self-seeding potency. We can quantify this effect by combining eqs 3 and 5,

5 $$k^{\prime }=k+2k_{\mathrm{frag}}k_{+}M_{\mathrm{seed}}\Delta t$$

If fragmentation is also the dominant secondary process in *de novo* fibril self-assembly, then κ^2^ = 2*k*_frag_ *k*_+_ *m*_original_, where *m*_original_ is the original monomer concentration used to assemble the seed fibrils before use in the seeded assay. This means there will be a direct link between κ and the effect of fragmentation on *k*,

6a $$k^{\prime }=k+\kappa^{2}r\Delta t$$

6b $$r=\frac{M_{\mathrm{seed}}}{m_{\mathrm{original}}}$$

However, eq 6 holds only if fragmentation is the dominant secondary process in *de novo* fibril assembly. If fragmentation is not the dominant secondary process, *k*′ will be smaller than the measured value of κ would predict, or conversely, κ will be too large to be explained by the change from *k* to *k*′ alone.

We globally fitted the fluorescence intensity changes over time from three replicates to an exponential decay,

7 $$y=a\left(1-e^{-\mathrm{kt}}\right)$$

where *y* is the normalized ThT intensity, *t* is time, *a* is the amplitude of the exponential fit, and *k* is the rate constant. eq 9 is simply a transformation of eq 3, and their rate constants have the same meaning. Theoretical predictions of *k*′ were then calculated using eq 6, where *k*′ is the predicted rate constant at *t* = 45 h if fragmentation were the dominant secondary process, κ is fitted from the corresponding *de novo* ThT assay, *r* is the seed dilution factor (0.1), and Δ*t* is the time interval between the first and second seeding reactions (i.e., 15 h). In all cases where this was performed, the observed *k*′ was much less than the predicted *k*′, indicating that fragmentation cannot be the dominant secondary process in *de novo* assembly.

### Mathematical Models of the Effect of Ionic Strength on **κ**

Extracted values of κ at varying ionic strengths were fitted to two mathematical models describing possible effects of ionic strength on the rate of secondary nucleation. We considered two possible scenarios: the “Free Energy Barrier” model, in which ions screen out an unfavorable electrostatic term in the free energy barrier for secondary nucleation; and the “Brønsted–Bjerrum” model, in which ions affect the rate by altering the activity of the precursor(s) of secondary nucleation. As described in (Derivations), the “Free Energy Barrier” model predicts saturation of κ at high ionic strength according to the relation,

8 $$\kappa =\kappa_{\mathrm{sat}}{\left(\frac{\kappa_{0}}{\kappa_{\mathrm{sat}}}\right)}^{2^{-\sqrt{I/I_{\mathrm{mid}}}}}$$

where κ_0_ and κ_sat_ are the limits of κ at low and high ionic strength, respectively, and *I*_mid_ is the ionic strength at which a midpoint is reached. Precise definitions are given in (Derivations). On the other hand, the “Brønsted–Bjerrum” model has a lower limit κ_0_ but no saturation,

$$\kappa =\kappa_{0}2^{\sqrt{I/I_{2}}}$$ 9

where *I*_2_ is the ionic strength at which there is a 2-fold enhancement of κ relative to κ_0_, and the definitions of κ_0_ and *I*_2_ are again given in (Derivations). Crucially, while eq 10 predicts the saturation of κ at high ionic strength, eq 11 does not.

The ‘Free Energy Barrier’ model is equivalent to stabilization of the critical nucleus of secondary nucleation, or an equivalent charged transition state in fragmentation, by electric field screening according to Debye–Hückel theory. The “Brønsted–Bjerrum” model is equivalent to stabilization of a secondary nucleation intermediate prior to the critical nucleus, i.e., reduced electrostatic repulsion between the monomers and the fibril surface.

To compare the ability of the models to describe the variation of κ with ionic strength, both models were fitted to values of κ extracted using eq 2 at different ionic strengths, and the quality of the fits was assessed using Akaike’s corrected information criterion (AICc). For the “Free Energy Barrier” model, the fitted parameters were κ_0_, κ_sat_, and *I*_mid_. For the "Brønsted–Bjerrum" model, the fitted parameters were κ_0_ and *I*_2_. Fitting was performed in GraphPad Prism 10.4.2 using Levenberg–Marquardt nonlinear least-squares regression.

### Pelleting Assay

A pelleting assay was used to determine the percentage of protein converted into insoluble material at the end of the ThT assays. Immediately following the end of the ThT assays, samples were retrieved from the assay plates and centrifuged at 100,000*g* for 30 min at 4 °C. The supernatant and whole fractions were loaded onto 15% Tris-tricine SDS-PAGE gels. The gels were subsequently stained with InstantBlue Coomassie stain, and the densitometry of the bands was measured using Nine-Alliance software. The percentage of pelletable material was determined using the equation

10 $$\mathrm{percentage}\;\mathrm{pelletable}=100\times 1-\left(\frac{D_{\mathrm{sol}}}{D_{\mathrm{whole}}}\right)$$

where *D*_sol_ is the densitometry of the soluble fraction and *D*_whole_ is the densitometry of the whole sample. The pelleting assay was performed three times.

### Negative Stain Transmission Electron Microscopy

Negative stain transmission electron microscopy (TEM) was carried out on the end products of the ThT assays. The samples were administered to carbon-coated copper grids, which were subsequently washed three times with 18 MΩ H_2_O and stained with 2% (*w/v*) uranyl acetate. Imaging was performed with a FEI Tecnai T12 electron microscope.

### Flow-Induced Dispersion Analysis (FIDA)

Flow-induced dispersion analysis (FIDA) was performed on a Fida-1 instrument (FidaBio) with a 75 μm × 1 m capillary. The capillary was washed with 1 M NaOH, then distilled water, then coated with HS reagent (FidaBio), with a final wash with distilled water. Monomeric protein was dissolved in 20 mM sodium phosphate buffer at pH 7.4 in the absence of NaCl, at a protein concentration of 400 μM. Analysis runs were carried out in the same buffer with the desired concentration of NaCl, as the small plug of the sample (∼50 nL) rapidly disperses into the NaCl-containing buffer after injection onto the capillary. Each FIDA run had 3 steps: (1) equilibration with buffer for 90 s at 3500 mbar; (2) injection of a plug of sample for 10 s at 50 mbar; and (3) elution with a further blank for 3 min at 400 mbar. The elution of protein was monitored by intrinsic fluorescence (excitation 275 nm, emission 300–450 nm), and the hydrodynamic radius was calculated from the baseline-subtracted Taylorgram using the FIDA analysis software (FidaBio).

### Molecular Dynamics (MD) Simulations

All coarse-grained MD simulations were performed using the CALVADOS 2 force field.^57,58^ Simulations were performed as stated previously,^57,58^ except for differences in box size and simulation duration as stated below. In short, CALVADOS uses a simplified representation of one bead per residue, connected by harmonic bonds with an equilibrium distance of 0.38 nm and a force constant of 8033 kJ mol^–1^·nm^–2^. Molecular interactions between nonadjacent beads are accounted for by additional potentials: a truncated and shifted Ashbaugh-Hatch potential for nonelectrostatic interactions, and a truncated Debye–Hückel potential for electrostatic interactions. Ionic strength is accounted for by changes in the Debye length used to model electrostatic interactions.^57,58^ At the start of each simulation, a single αSyn monomer was initialized as a linear polymer with beads separated by 0.38 nm in a periodic box of size 0.76­(N-1) + 4 nm, where N is the number of beads. Simulations were carried out using a Langevin integrator with a time step of 10 fs and a friction coefficient of 0.01 ps^–1^, with a sampling frequency of 70 ps (7000 timesteps) per frame, which yields weakly correlated frames.^58^ After an initial equilibration of 700 ps (7 × 10^4^ timesteps), the simulation was carried out for 350 ns (35 × 10^6^ timesteps) to obtain 5000 frames sampling the protein’s simulated conformational landscape. All simulations were carried out at 310 K and pH 7.4 at the ionic strengths indicated in the text. The structural parameters *R*_g_, ν, Δ, and S and their errors were calculated as described in.^58^

To classify αSyn conformers by spectral clustering, we first calculated the pairwise similarity (affinity) of each pair of frames in a simulation. We chose to compare inter-residue C_α_–C_α_ distances rather than aligned C_α_ coordinates, as inter-residue distances preserve information about structural contacts in spite of the large continuous deformations typically seen in CALVADOS 2 simulations of IDRs. The affinity score A_*xy*_ for each pair of frames (*x*, *y*) was calculated as the Gaussian kernel of the mean squared deviation in inter-residue (C_α_–C_α_) distances between the two frames, using the mean expected squared deviation in inter-residue distances as the normalization. Specifically,

11 $$A_{\mathrm{xy}}=\exp\left[-\frac{\left\langle \Delta d_{\mathrm{ij}}^{2}\right\rangle }{\left\langle E\left(\Delta d_{\mathrm{ij}}^{2}\right)\right\rangle }\right]$$

where ⟨···⟩ = ∑_*i*>*j*+1_···/*N* represents an average across all *N* nonbonded residue pairs (*i*, *j*), Δ*d*_*ij*_^2^ = (*d*_*ij*,*x*_ – *d*_*ij*,*y*_)^2^ is the squared deviation in inter-residue distance *d*_*ij*_ between frames *x* and *y*, and $E\left(\Delta d_{\mathrm{ij}}^{2}\right)=2\mathrm{Var}\left(d_{\mathrm{ij}}\right)$ is the expected squared deviation in *d*_*ij*_ for a randomly chosen pair of independent and identically distributed frames. Note that the mean across residue pairs ⟨···⟩ was taken separately on the numerator and denominator to preserve a stronger weighting for residue pairs that experience large variations throughout the simulation trajectory. As a result, *A*_*xy*_ is essentially a Gaussian transformation of the Euclidean distance between frames in the higher-dimensional (*d*_13_···*d*_*R*–2,*R*_) space, for a sequence of *R* residues. We then used the resulting affinity matrix **A** to classify frames into 4 clusters using the SpectralClustering class in scikit-learn.^93^ Alternative numbers of clusters provided similar results, but were less interpretable as they failed to separately resolve intradomain and long-range interactions. Note that we do not claim that these clusters represent distinct basins in the conformational free energy landscape of αSyn, which appears to be continuous in the CALVADOS 2 simulations. Instead, the spectral clustering algorithm provides a means to carve up the conformational spectrum of αSyn into closely related states. The increase in the population of the compact class with exon 5 present, therefore, likely reflects the skewing of the conformational distribution to produce an enhanced population of closely interrelated compact states. As clustering was carried out independently for each variant, the compact classes of different variants are not necessarily the same; however, inspection of the per-cluster contact maps showed the same pattern of N–C interactions in each case.

## Supplementary Material



## Data Availability

The data supporting the findings of this study are available from 10.5518/1695.

## Notes

Summary of mRNA expression levels of the alternative splice variants of αSyn in disease, *T*_50_ values, scaling exponents, AmyloFit fitted parameters, and insoluble protein yield for the ThT assay at different starting monomer concentrations, insoluble protein yields from cross-seeding experiment, sequence properties of the alternative splice variants of αSyn, fitted values (λ/κ/θ) and insoluble protein yield for the ionic strength ThT assay, comparison between the “Brønsted–Bjerrum” and “Free Energy Barrier” models, fitted values from the “Free Energy Barrier” model, *R*_h_ values of the alternative splice variants of αSyn at different added NaCl concentrations, CALVADOS 2 predicted *R*_g_ values, and chain-length-independent measures of the alternative splice variants of αSyn at different ionic strengths, primers used in this work; fitted models from AmyloFit, *de novo* amyloid kinetics of αSynΔ5 and αSynΔ3Δ5 at low starting monomer concentration, self-seeding kinetics, yield, and negative stain images, fitted curves for the ThT assays at different ionic strengths, fitted λ values, confidence ellipses from λ and κ fitting, elongation-dominated self-seeding at different points in the plateau phase, attempts to fit the κ values using the Brønsted–Bjerrum equation, representative Taylorgrams, contact probability maps, contact probability difference maps, spectral clustering probability distributions, proportion of clustering frames across ionic strengths, representative conformations with different Δ and S values, correlations of ranked data, Spearman’s rank heatmap of inter-residue distances, derivations of the “Free Energy Barrier” and “Brønsted–Bjerrum” models of the effect of ionic strength on κ (PDF)

## Abbreviations

AICc: Akaike’s corrected information criterion
αSyn: α-synuclein
αSynFL: α-synuclein full-length
CV: column volume
DLB: dementia with lewy bodies
FIDA: flow-induced dispersion analysis
IM-MS: ion mobility mass spectrometry
MD: molecular dynamics
MSA: multiple system atrophy
NAC: nonamyloid β component
PD: Parkinson’s disease
*R*_g_: radius of gyration
*R*_h_: hydrodynamic radius
SEM: standard error of the mean
TEM: transmission electron microscopy
ThT: thioflavin T
