# Supplementary material for: Mapping the Role of Monomer Conformation in the Amyloid Formation of α‑Synuclein Splice Variants
Source: J Am Chem Soc. 2025 Oct 24;147(44):40519–36. doi: 10.1021/jacs.5c12366 (PMC12593362; doi:10.1021/jacs.5c12366)
Supplement: Supplementary file 1 [file ja5c12366_si_001.pdf]

Supporting Information

Mapping the Role of Monomer Conformation in Amyloid Formation of α-synuclein Splice Variants

Katherine M. Dewison, Alexander I. P. Taylor, David J. Brockwell, Sheena E. Radford**

Astbury Centre for Structural Molecular Biology and School of Molecular and Cellular Biology, Faculty of Biological Sciences, University of Leeds, Leeds, LS2 9JT, UK

Supplementary Tables

Table S1. Changes in the mRNA levels of the alternative splice variants of *SNCA* compared with non-synucleinopathy controls.

Disease	N	Control N	Brain region	*SNCA-FL*	*SNCA-Δ3*	*SNCA-Δ5*	*SNCA-Δ3Δ5*	Ref.
**DLB**	6	6	Prefrontal cortex	Down*		Up***		1
**DLB**	6	10	Prefrontal cortex		Down***			2
**PD**	5	10	Frontal cortex	Up*	Up***	Up*	Up***	3
**DLBp**	6	10	Frontal cortex	Down***	Down***	Up*	Up***	3
**cLBD**	8	10	Frontal cortex	Similar	Down***	Down*	Up*	3
**AD**	8	10	Frontal cortex	Similar	Down***	Down*	Up*	3
**MSA**	6	10	Frontal cortex		Similar		Up**	3
**PD**	6	5	Substantia nigra	Up*	Up*	Similar	Similar	4
**PD**	6	6	Frontal cortex	Similar	Similar	Similar	Similar	4
**PD**	6	6	Cerebellum	Similar	Similar	Similar	Similar	4
**PD**	9	6	Substantia nigra	Similar	Similar	Similar	Similar	5
**PD**	9	6	Cerebellum	Similar	Similar	Up*	Up*	5
**PD**	9	6	Occipital cortex	Similar	Similar	Similar	Similar	5
**MSA**	12	12	Substantia nigra	Up***	Down***	Up***	Similar	6
**MSA**	12	12	Striatum	Up**	Down***	Up***	Similar	6
**MSA**	12	12	Cerebellar cortex	Up**	Down*	Up***	Similar	6
**MSA**	12	12	Dentate nucleus	Up***	Down*	Up***	Similar	6
**MSA**	12	12	Prefrontal cortex	Similar	Down*	Up*	Up*	6
**PD**	20	12	Striatum	Similar	Down***	Similar	Similar	6
**PD**	20	12	Cerebellar cortex	Up***	Down**	Similar	Similar	6
**PD**	20	12	Prefrontal cortex	Similar	Similar	Similar	Up*	6

Dementia with Lewy bodies (DLB), Parkinson's disease (PD), pure form of DLB (DLBp), common Lewy body disease (cLBD), and multiple system atrophy (MSA). N = number of post-mortem brain tissue samples used. Control N = number of compared control samples. The results on whether each variant was upregulated (green), downregulated (red), or similarly

expressed (yellow) between the disease tissue and the control tissue is displayed. * = $p < 0.05$, ** = $p < 0.01$, and *** = $p < 0.001$.

Table S2. T_{50} of amyloid formation of the alternative splice variants of αSyn at concentrations ranging from 20 µM to 100 µM.

Variant	Concentration (µM)	T_{50} (h)
**αSynFL**	100	7.8 ± 0.4
	80	9.8 ± 1.4
	60	12.2 ± 1.1
	40	14.5 ± 1.4
	20	20.8 ± 1.4
**αSynΔ3**	100	10.0 ± 0.4
	80	11.1 ± 0.4
	60	12.6 ± 0.6
	40	14.9 ± 0.9
	20	21.7 ± 1.4
**αSynΔ5**	100	5.9 ± 0.3
	80	6.1 ± 0.4
	60	5.4 ± 0.3
	40	4.9 ± 0.3
	20	4.8 ± 0.4
**αSynΔ3Δ5**	100	4.1 ± 0.6
	80	4.3 ± 0.6
	60	5.2 ± 0.8
	40	3.7 ± 0.3
	20	4.9 ± 0.5

Error is SEM.

Table S3. Scaling exponents determined from the T_{50} values and starting concentration of monomer.

Variant	Scaling exponent	95% confidence interval
**αSynFL**	-0.55	-0.68 to -0.43
**αSynΔ3**	-0.49	-0.57 to -0.40
**αSynΔ5**	0.17	0.057 to 0.28
**αSynΔ3Δ5**	-0.064	-0.27 to 0.15

Table S4. Fitted parameters from the four tested models in AmyloFit[7] for αSynFL and αSynΔ3.

		**αSynFL**	**αSynΔ3**
**Elongation dominated, unseeded**	Mean residual error	0.0186	0.0148
	k_+k_n ($M^{-nc}.s^{-2}$)	6.51e-5	5.52e-6
	n_c	1.17	0.984
**Secondary nucleation dominated, unseeded**	Mean residual error	0.0132	0.00454
	k_+k_n ($M^{-nc}.s^{-2}$)	2.24e-10	1.04e-10
	n_c	0.0847	0.0000214
	k_+k_2 ($M^{-n2-1}.s^{-2}$)	17.5	6.23e4
	n_2	1.17	2.21
**Fragmentation dominated, unseeded**	Mean residual error	0.0134	0.00500
	k_+k_n ($M^{-nc}.s^{-2}$)	3.21e-4	2.77e-6
	n_c	1.43	1.04
	k_+k_- ($M^{-1}.s^{-2}$)	4.95	3.58e-5
**Multi-step secondary nucleation dominated, unseeded**	Mean residual error	0.0132	0.00344
	k_+k_n ($M^{-nc}.s^{-2}$)	1.76e-10	7.42e-11
	n_c	0.0608	0.0000153
	k_+k_2 ($M^{-n2-1}.s^{-2}$)	78.7	13.1
	n_2	1.32	1.29
	K_M (μM^{n2})	33.1	1860

Table S5. Yield of insoluble material following *de novo* fibril formation reactions of the alternative splice variants of αSyn at concentrations ranging from 20 µM to 100 µM.

Variant	Concentration (µM)	Insoluble (%)
**αSynFL**	100	90 ± 0
	80	87 ± 2
	60	85 ± 0
	40	82 ± 2
	20	77 ± 4
**αSynΔ3**	100	95 ± 0
	80	92 ± 2
	60	90 ± 0
	40	78 ± 7
	20	63 ± 4
**αSynΔ5**	100	92 ± 2
	80	90 ± 3
	60	90 ± 3
	40	92 ± 2
	20	85 ± 3
**αSynΔ3Δ5**	100	90 ± 3
	80	88 ± 2
	60	88 ± 3
	40	88 ± 3
	20	70 ± 6

Error is SEM.

Table S6. Yield of insoluble protein resulting from cross-seeding ThT assays of αSynFL monomers with the alternative splice variant fibril seeds.

Variant seed	Insoluble (%)
**αSynΔ3**	6 ± 17
**αSynΔ5**	72 ± 11
**αSynΔ3Δ5**	20 ± 18

Note that the monomer used for all experiments was αSynFL and the identity of the fibril seed is indicated in each row. Error is SEM.

Table S7. Sequence properties of the alternative splice variants of αSyn.

Variant	Sequence hydropathy decoration[8]	Sequence charge decoration[8,9]	κ (charge patterning)[10]	Fraction of charged residues	Net charge per residue
**αSynFL**	3.081	-2.110	0.168	0.293	-0.064
**αSynΔ3**	2.990	-1.267	0.172	0.302	-0.079
**αSynΔ5**	2.992	-0.994	0.105	0.227	0.009
**αSynΔ3Δ5**	2.885	-0.951	0.108	0.286	0.000

Table S8. Fitted values of the alternative splice variants of αSyn for the rate of the primary (λ) and secondary pathways (κ), and θ at [NaCl] ranging from 0 mM to 400 mM.

Variant	[NaCl] (mM)	λ ($hour^{-1}$)	κ ($hour^{-1}$)	θ (no units)
**αSynFL**	0	0.038 ± 0.008	0.590 ± 0.079	0.540
	50	0.008 ± 0.003	1.104 ± 0.101	0.540
	100	0.010 ± 0.005	1.269 ± 0.130	0.540
	200	0.001 ± 0.001	2.170 ± 0.161	0.540
	300	0.002 ± 0.001	2.300 ± 0.193	0.540
	400	0.002 ± 0.001	2.113 ± 0.201	0.540
**αSynΔ3**	0	-	-	-
	50	0.038 ± 0.002	0.361 ± 0.018	0.568
	100	0.034 ± 0.003	0.629 ± 0.036	0.568
	200	0.046 ± 0.004	0.919 ± 0.035	0.568
	300	0.026 ± 0.005	1.550 ± 0.064	0.568
	400	0.015 ± 0.004	2.062 ± 0.082	0.568
**αSynΔ5**	0	0.048 ± 0.008	0.705 ± 0.074	3.000
	50	0.046 ± 0.019	1.386 ± 0.075	3.000
	100	0.064 ± 0.026	1.944 ± 0.096	3.000
	200	0.039 ± 0.016	2.418 ± 0.121	3.000
	300	0.112 ± 0.034	2.472 ± 0.143	3.000
	400	0.088 ± 0.025	2.640 ± 0.190	3.000
**αSynΔ3Δ5**	0	0.125 ± 0.020	1.458 ± 0.102	2.657
	50	0.155 ± 0.019	3.223 ± 0.331	2.657
	100	0.133 ± 0.031	4.771 ± 0.302	2.657
	200	0.172 ± 0.036	5.143 ± 0.401	2.657
	300	0.257 ± 0.029	5.395 ± 0.121	2.657
	400	0.278 ± 0.028	5.217 ± 0.118	2.657

Note that θ describes how the rate of new fibril formation depends on the monomer concentration. Error is SEM.

Table S9. Percent insoluble material formed by the end of the ThT assays of the alternative splice variants of αSyn in the presence of different concentrations of NaCl.

Variant	[NaCl] (mM)	Insoluble (%)
**αSynFL**	0	74 ± 1
	50	81 ± 1
	100	86 ± 3
	200	90 ± 1
	300	94 ± 2
	400	96 ± 2
**αSynΔ3**	0	1 ± 5
	50	87 ± 2
	100	94 ± 0
	200	94 ± 2
	300	96 ± 1
	400	98 ± 1
**αSynΔ5**	0	91 ± 1
	50	91 ± 2
	100	93 ± 1
	200	96 ± 0
	300	98 ± 1
	400	98 ± 0
**αSynΔ3Δ5**	0	93 ± 2
	50	95 ± 1
	100	96 ± 0
	200	97 ± 1
	300	97 ± 1
	400	98 ± 0

Error is SEM.

Table S10. Comparison between the 'Brønsted-Bjerrum' and 'Free Energy Barrier' models of behaviour of the variants in terms of rate of secondary pathway of amyloid formation at different ionic strength.

	**Brønsted-Bjerrum**	**Free Energy Barrier**	**Preferred model**	**Difference in AICc**
**αSynFL**	0.7768%	99.22%	Free Energy Barrier	9.700
**αSynΔ3**	37.16%	62.84%	Free Energy Barrier	1.051
**αSynΔ5**	<0.01%	>99.99%	Free Energy Barrier	30.64
**αSynΔ3Δ5**	<0.01%	>99.99%	Free Energy Barrier	53.55

Akaike's corrected Information Criterion (AICc) was used to determine the preferred model (Experimental Section).

Table S11. Fitted values from the 'Free Energy Barrier' model of the influence of ionic strength on the rate of the secondary pathway of amyloid formation.

	**κ_0 (hour^{-1})**	**κ_{sat} (hour^{-1})**	**I_{mid} (mM)**	**$\Delta\Delta G^{\ddagger}_{non\text{-}charged}$ (RT)**
**αSynFL**	0.017 ± 0.027	2.66 ± 0.43	16.2 ± 9.2	N/A
**αSynΔ3**	0.033 ± 0.022	25.53 ± 45.11	230.6 ± 247.5	-4.52 ± 3.55
**αSynΔ5**	0.0014 ± 0.0028	2.75 ± 0.18	8.0 ± 2.6	-0.07 ± 0.35
**αSynΔ3Δ5**	1.7×10^{-6} ± 9.3×10^{-6}	5.47 ± 0.26	4.0 ± 1.2	-1.44 ± 0.34

The model accounts for electrostatic and non-electrostatic contributions to the free energy barrier between the alternative splice variants. The fitted parameters are: κ_0, the limit of κ at low ionic strength; κ_{sat}, the limit of κ at high ionic strength; and I_{mid}, the ionic strength at which κ is halfway between its upper and lower limits on a logarithmic scale. Based on κ_{sat}, we calculated $\Delta\Delta G^{\ddagger}_{non\text{-}charged}$, the change in the non-electrostatic contribution to the free energy barrier. In this table, the $\Delta\Delta G^{\ddagger}_{non\text{-}charged}$ are given relative to αSynFL, but we also considered the difference in barrier height between αSynΔ3Δ5 and αSynΔ5 ($\Delta\Delta G^{\ddagger}_{non\text{-}charged}$ = -1.38 ± 0.16 RT) in the main text. Note that, due to the practical difficulties with obtaining sufficiently low-concentration data, it was not possible to precisely determine κ_0, but this does not affect fitting of κ_{sat}, which is the sole parameter needed to determine $\Delta\Delta G^{\ddagger}_{non\text{-}charged}$. For derivation of the model and definitions of κ_0, κ_{sat} and I_{mid} in terms of free energy, see Supplemental Derivations.

Table S12. R_h values of the alternative splice variants of αSyn at different added NaCl concentrations and the predicted R_h of an unfolded protein of equivalent chain length.

	R_h (nm)						
**Variant**	Predicted unfolded	0 mM NaCl	50 mM NaCl	100 mM NaCl	200 mM NaCl	300 mM NaCl	400 mM NaCl
**αSynFL**	3.70	3.05 ± 0.02	3.18 ± 0.03	3.20 ± 0.02	3.24 ± 0.02	3.27 ± 0.02	3.28 ± 0.03
**αSynΔ3**	3.48	2.87 ± 0.03	3.02 ± 0.04	3.10 ± 0.04	3.11 ± 0.04	3.15 ± 0.03	3.15 ± 0.03
**αSynΔ5**	3.25	2.88 ± 0.01	2.89 ± 0.01	2.90 ± 0.01	2.91 ± 0.003	2.92 ± 0.01	2.91 ± 0.02
**αSynΔ3Δ5**	3.02	2.63 ± 0.01	2.63 ± 0.01	2.64 ± 0.01	2.65 ± 0.01	2.66 ± 0.01	2.66 ± 0.02

R_h measured by FIDA and fitted in the FidaBio software. Error is SEM. Predicted R_h of unfolded polypeptide chain was calculated based on a previously derived equation[11] (Experimental Section).

Table S13. R_g values of the alternative splice variants of αSyn at different ionic strengths as calculated from the CALVADOS 2 simulations.

	R_g (nm)					
**Variant**	0 mM NaCl	50 mM NaCl	100 mM NaCl	200 mM NaCl	300 mM NaCl	400 mM NaCl
**αSynFL**	3.42 ± 0.03	3.63 ± 0.02	3.70 ± 0.02	3.74 ± 0.02	3.76 ± 0.02	3.76 ± 0.02
**αSynΔ3**	3.30 ± 0.02	3.46 ± 0.02	3.50 ± 0.02	3.54 ± 0.02	3.55 ± 0.02	3.57 ± 0.02
**αSynΔ5**	3.23 ± 0.01	3.25 ± 0.01	3.28 ± 0.01	3.29 ± 0.01	3.28 ± 0.01	3.30 ± 0.01
**αSynΔ3Δ5**	2.98 ± 0.01	3.02 ± 0.01	3.02 ± 0.01	3.03 ± 0.01	3.05 ± 0.01	3.05 ± 0.01

Error is SEM.

Table S14. Chain-length independent measures of the compaction and shape of the alternative splice variants under different ionic strengths where Δ is asphericity, S is prolateness and ν is the Flory exponent.

Variant	[NaCl] (mM)	Δ	S	ν
**αSynFL**	0	0.142 ± 0.003	0.404 ± 0.010	0.463 ± 0.004
	50	0.158 ± 0.003	0.472 ± 0.011	0.524 ± 0.002
	100	0.163 ± 0.003	0.489 ± 0.009	0.539 ± 0.001
	200	0.167 ± 0.003	0.509 ± 0.010	0.545 ± 0.002
	300	0.170 ± 0.003	0.531 ± 0.010	0.555 ± 0.001
	400	0.170 ± 0.003	0.530 ± 0.010	0.554 ± 0.001
**αSynΔ3**	0	0.153 ± 0.003	0.422 ± 0.010	0.488 ± 0003
	50	0.156 ± 0.003	0.476 ± 0.008	0.533 ± 0.002
	100	0.167 ± 0.003	0.495 ± 0.009	0.543 ± 0.002
	200	0.173 ± 0.003	0.527 ± 0.009	0.556 ± 0.001
	300	0.175 ± 0.003	0.538 ± 0.008	0.560 ± 0.001
	400	0.173 ± 0.003	0.545 ± 0.009	0.561 ± 0.001
**αSynΔ5**	0	0.168 ± 0.003	0.528 ± 0.009	0.539 ± 0.003
	50	0.174 ± 0.003	0.540 ± 0.008	0.546 ± 0.003
	100	0.171 ± 0.003	0.553 ± 0.008	0.553 ± 0.002
	200	0.177 ± 0.003	0.555 ± 0.008	0.558 ± 0.002
	300	0.172 ± 0.003	0.547 ± 0.008	0.553 ± 0.002
	400	0.176 ± 0.003	0.555 ± 0.009	0.564 ± 0.002
**αSynΔ3Δ5**	0	0.172 ± 0.003	0.530 ± 0.008	0.537 ± 0.003
	50	0.174 ± 0.002	0.551 ± 0.008	0.553 ± 0.003
	100	0.175 ± 0.002	0.544 ± 0.008	0.547 ± 0.003
	200	0.178 ± 0.003	0.552 ± 0.007	0.557 ± 0.002
	300	0.172 ± 0.002	0.555 ± 0.007	0.556 ± 0.003
	400	0.174 ± 0.003	0.558 ± 0.008	0.558 ± 0.003

As calculated based on the CALVADOS 2 simulations. The error for Δ and S are SEM, while the error for the ν is the fitting error.

Table S15. DNA sequence of the primers used to delete exons 3 (residues 41 to 54) and/or 5 (residues 103 to 130) from the pET23a vector encoding αSynFL (Experimental Section).

	Forward	Reverse
**Δ3**	GTTGCGGAGAAAACGAAAG	GACATACAGCACACCTTC
**Δ5**	GAAGGCTACCAGGACTAC	TTTCCCTAACTGGTCTTTC

Supplementary Figures

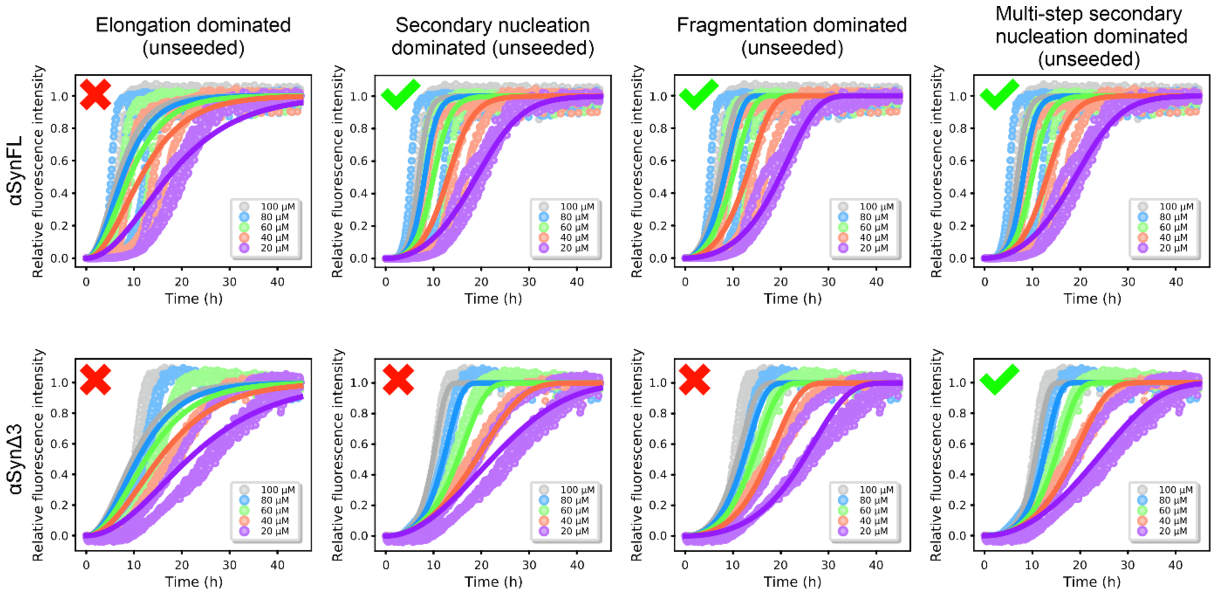

Figure S1. Normalised ThT fluorescence curves for αSynFL (top) and αSynΔ3 (bottom) from Figure 2a with the fitted models from AmyloFit[7]. The four fitted models used were 'Elongation dominated (unseeded)', 'Secondary nucleation dominated (unseeded)', 'Fragmentation dominated (unseeded)', and 'Multi-step secondary nucleation dominated (unseeded)'. The fitted curves are plotted as the solid lines. The favoured models are indicated with a green tick, whereas the unfavoured models are indicated with a red cross. Favoured models are defined as those with the lowest mean residual error (Table S4).

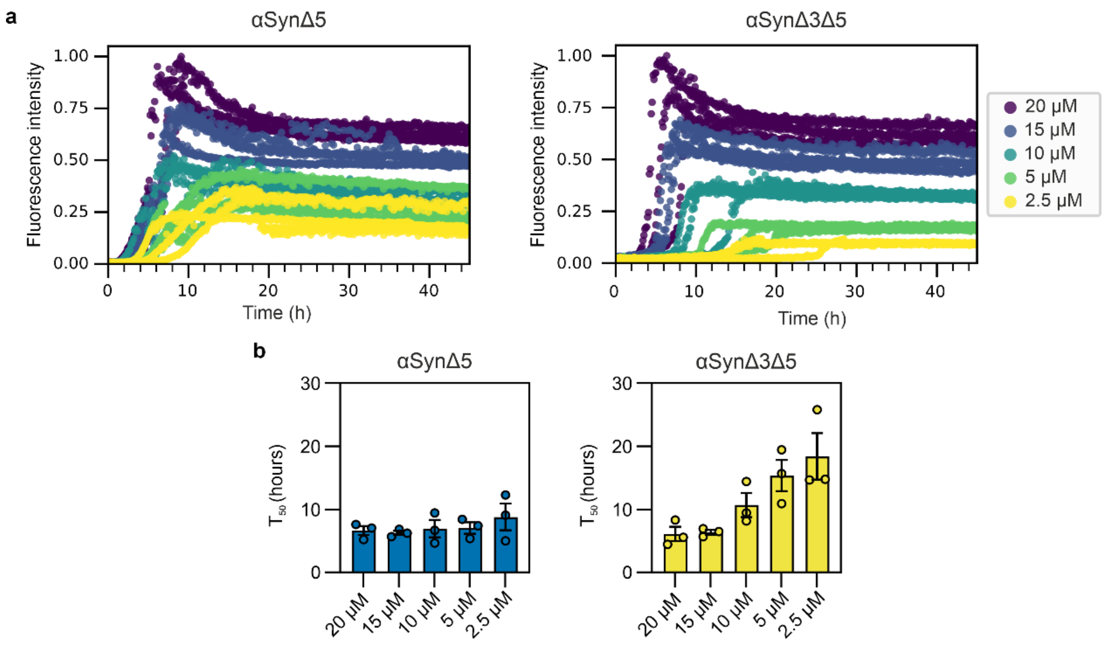

Figure S2. *De novo* fibril formation assays for αSynΔ5 and αSynΔ3Δ5 at initial starting monomer concentrations of 2.5 μM to 20 μM. (a) ThT fluorescence traces of αSynΔ5 and αSynΔ3Δ5. Data are normalised to the maximum fluorescence intensity of each variant at 20 μM αSyn concentration. (b) T_{50} values of each reaction, where datapoints represent the calculated T_{50} value of each replicate, and the error bar is SEM.

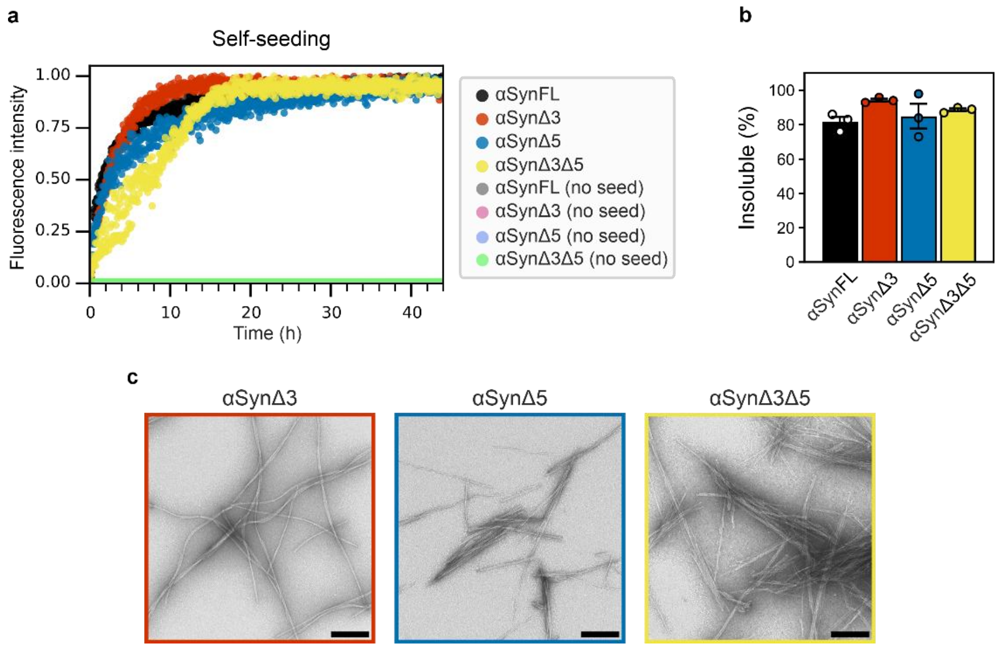

Figure S3. Self-seeding of the alternative splice variants of αSyn. (a) Representative ThT kinetics of self-seeding of αSynFL, αSynΔ3, αSynΔ5, and αSynΔ3Δ5. Note that there was no increase in ThT fluorescence for any of the 'no seed' conditions. (b) Percentage of insoluble material generated at the end point of the self-seeding reactions. Each datapoint indicates the percent insoluble material formed by one of the three repeats. Error bars are SEM. (c) Negative stain TEM of the material formed during the self-seeding ThT assays. TEM images of self-seeded αSynFL are shown in Figure 3c. Scale bar, 250 nm.

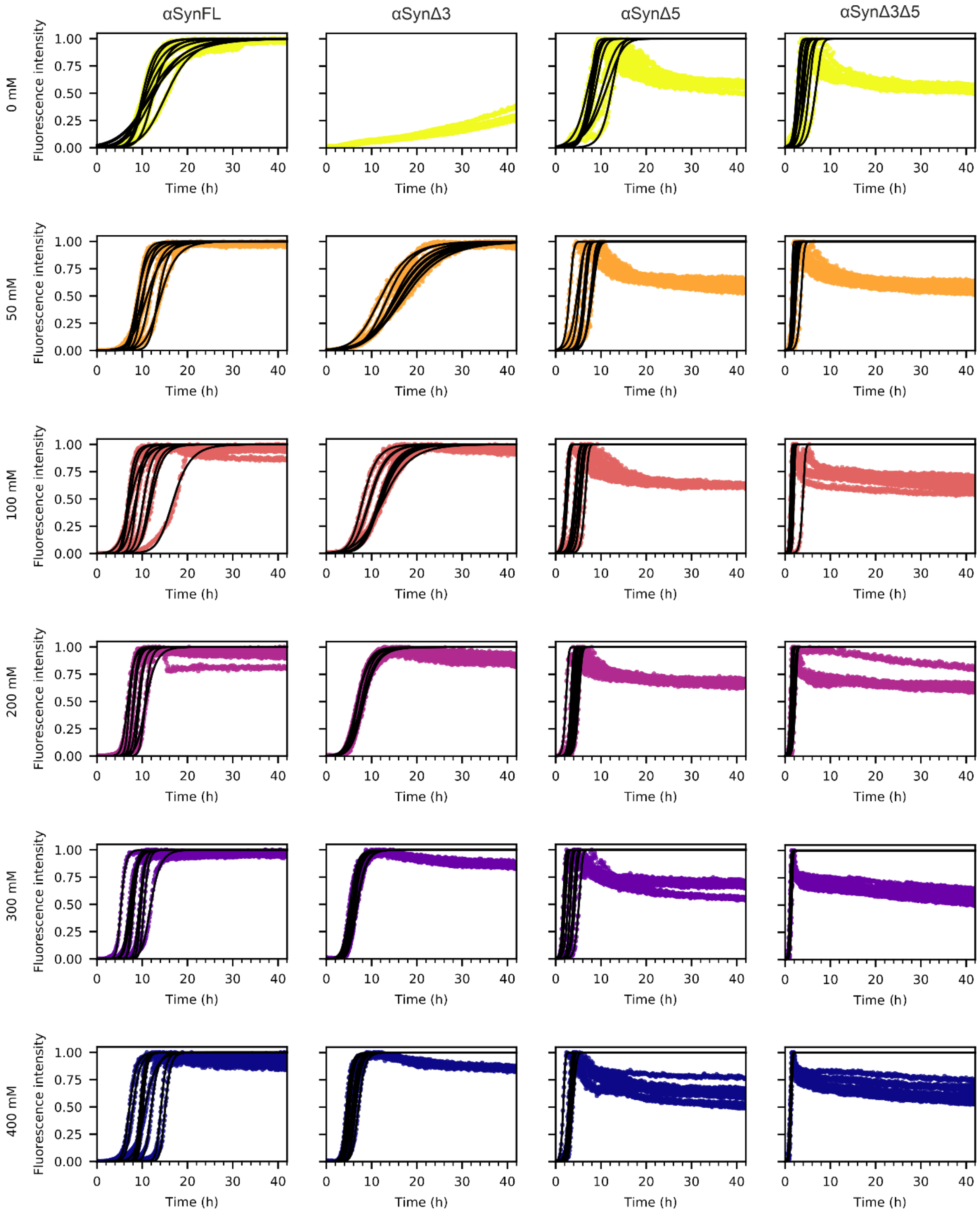

Figure S4. Fitted curves for the ThT assays with the splice variants carried out at different ionic strengths. The fitted curves are shown in black. Identities of isoform and NaCl concentration are indicated at the top of columns and left of rows, respectively. Curves were fitted using equation [2t] derived previously[12] (Experimental Section). Note that only the data

before the maximum signal were considered for fitting to avoid skewing of the data by the decrease in ThT fluorescence observed at longer timescales, particularly for αSynΔ5 and αSynΔ3Δ5. This decrease in signal is hypothesised to be due to flocculation of amyloid fibrils and is not accounted for in current models of amyloid assembly. θ values were fitted globally for each variant and constrained to be <3.

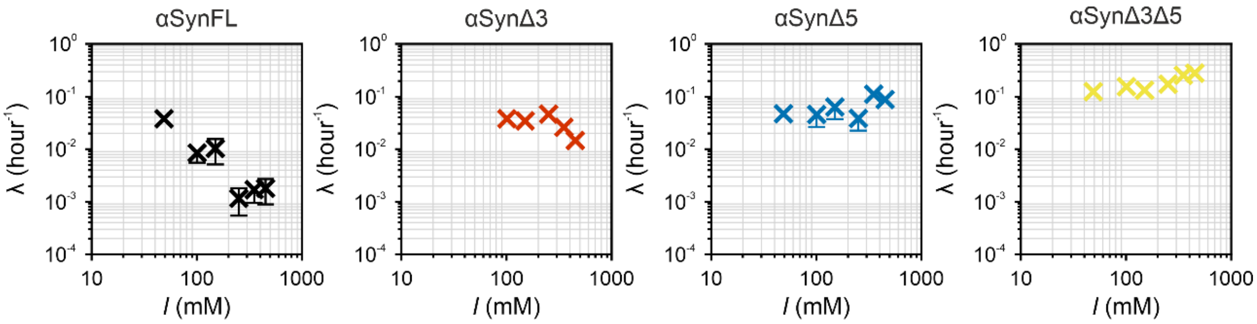

Figure S5. λ values for the splice variants of αSyn across a range of ionic strengths. λ values were derived from the fitted curves shown in Figure S4. Error bars are SEM. Note that in some cases the error bars could not be plotted because they are shorter than the size of the symbol. The plotted ionic strength (*I*) is that of the total buffer.

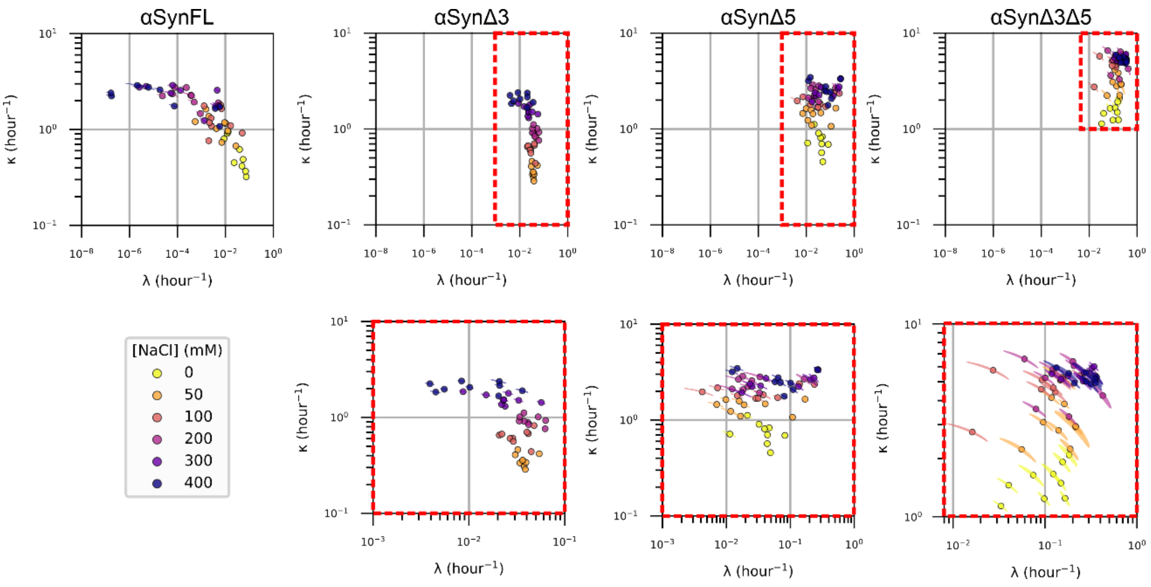

Figure S6. The fitted λ and κ values for individual replicates of the kinetics shown in Figure S4. Individual plots are used for different splice variants (indicated above each plot) and the datapoints are coloured by added [NaCl] (mM) (indicated in key). The red dashed lines indicate the areas magnified in the plots beneath. Each circle represents the best-fit values from equation [1]. Surrounding each point is a 95% confidence ellipse, calculated from the covariance matrix of the parameter estimates. These ellipses represent the joint uncertainty of λ and κ. In most cases, the size of the confidence ellipse is smaller than the inter-replicate variability that arises from well-to-well differences.

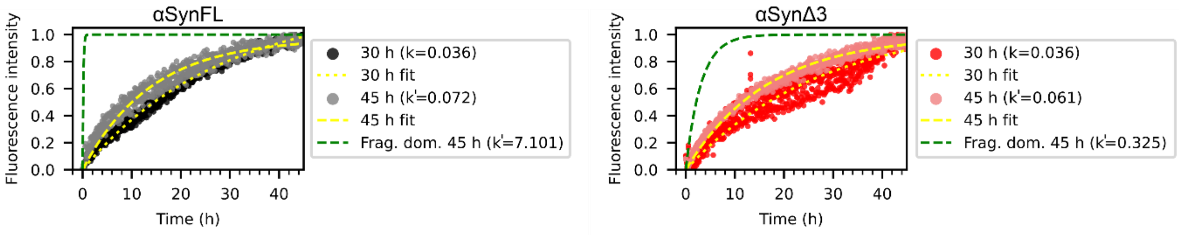

Figure S7. Self-seeding using seeds from different points of the plateau phase. An initial *de novo* ThT assay was set up for αSynFL and αSynΔ3 (20 mM sodium phosphate, 100 mM NaCl, pH 7.4) (Experimental Section). Fibrils were collected from the plateau phase at either 30 h or 45 h and incubated with monomer of the same variant (50 µM monomer plus 10 µM fibril seeds (monomer equivalent concentration)). Data were fitted using Eq. 7 to determine the rate constant, k. The value of k from the seeding data using seeds from 30 h was used to predict the rate constant of seeds after 45 h, k', that would be expected if fragmentation were the dominant secondary process in our experimental conditions using Eq. 6 (green dashed line).

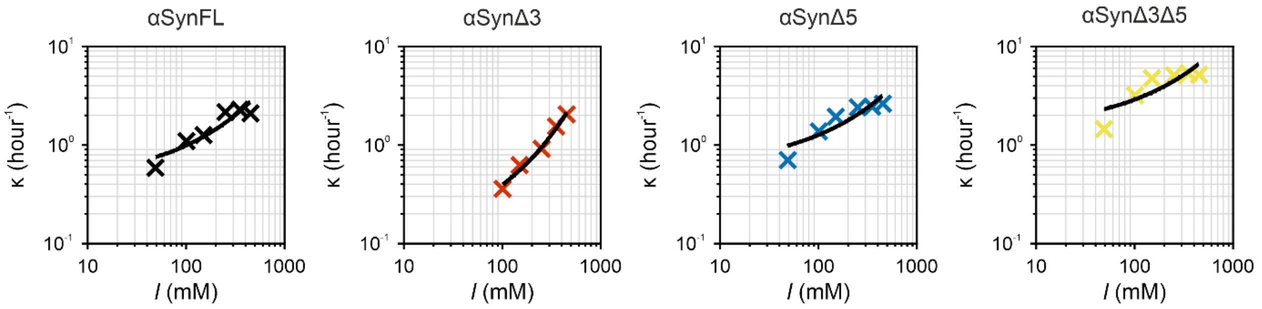

Figure S8. κ values derived from the fitted kinetic analysis (see Figure S4). The data are fitted here using the 'Brønsted-Bjerrum' equation (Experimental Section). The plotted ionic strength (*I*) is that of the total buffer.

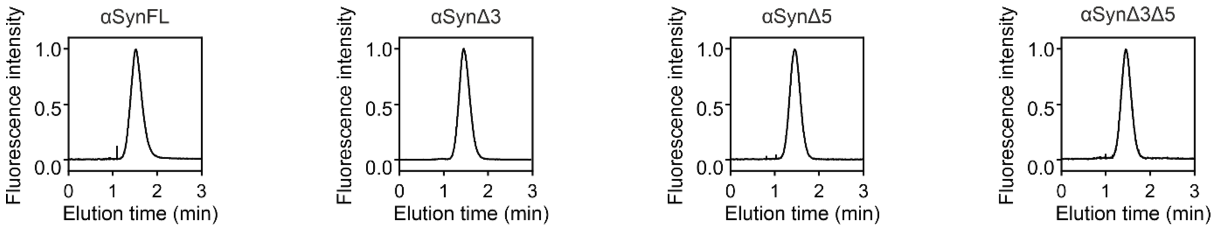

Figure S9. Representative Taylorgrams of the alternative splice variants of αSyn collected at 0 mM NaCl. Fluorescence intensity is normalised to the maximum intensity of the Taylorgram.

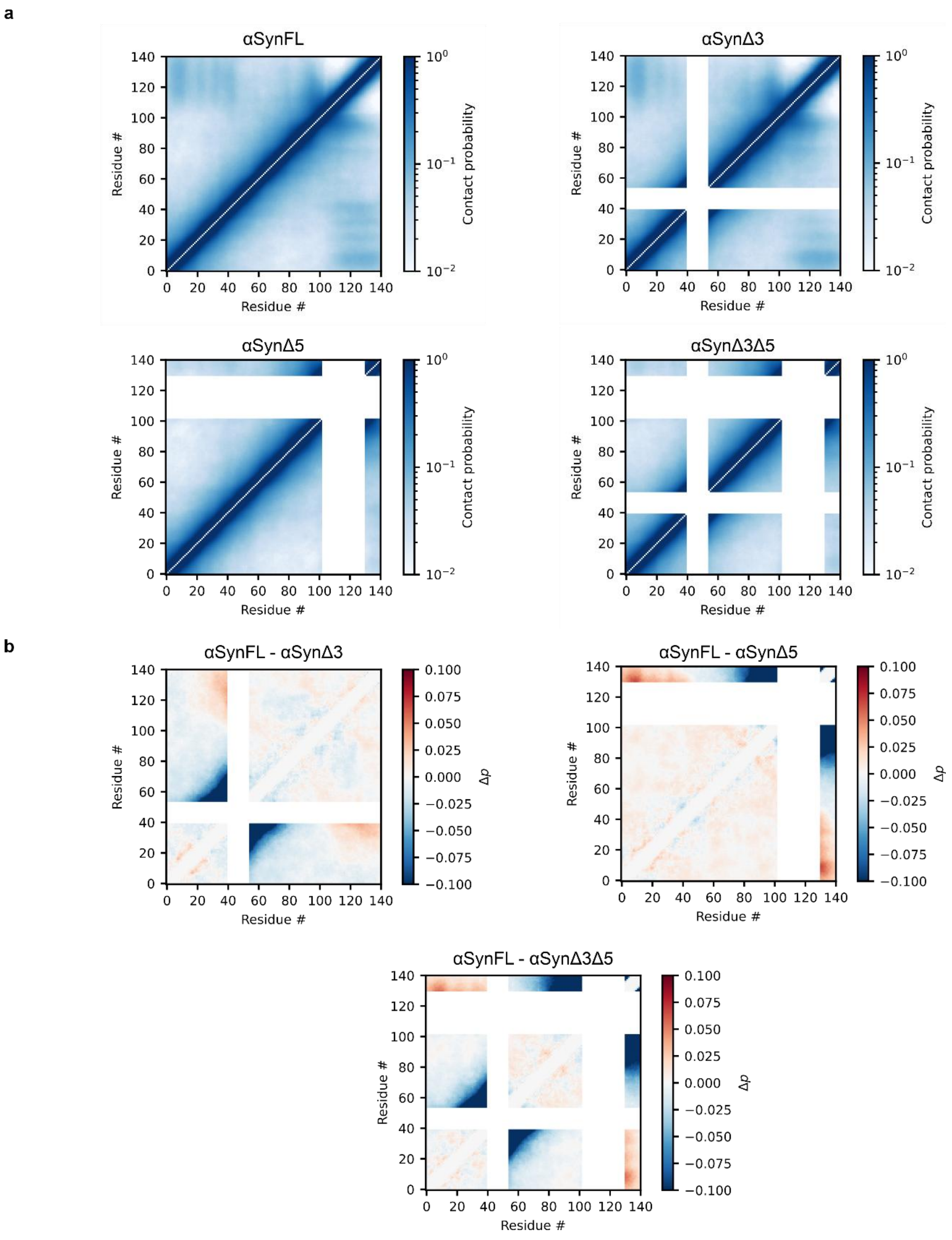

Figure S10. Contact probability maps for αSynFL, αSynΔ3, αSynΔ5, and αSynΔ3Δ5. (a) Contact probability maps for each variant at 49 mM ionic strength (equivalent to the lowest ionic strength, 0 mM NaCl, used in FIDA and ThT experiments) generated from the CALVADOS 2 simulations using a 20 Å cutoff. (b) Contact probability differences (Δp)

calculated by summing the contacts (<20 Å) for each residue pair and subtracting that of the alternative splice variant with padding to account for deleted regions, such that residue pairs with a positive (red) Δp are predicted to interact more frequently in the αSynFL variant, and negative (blue) Δp values represent residue pairs that more frequently interact in the compared variant. Note that as residues that are closer together in the primary sequence have an inherent propensity to interact, this causes regions close to the sites of deletion to have a more negative Δp.

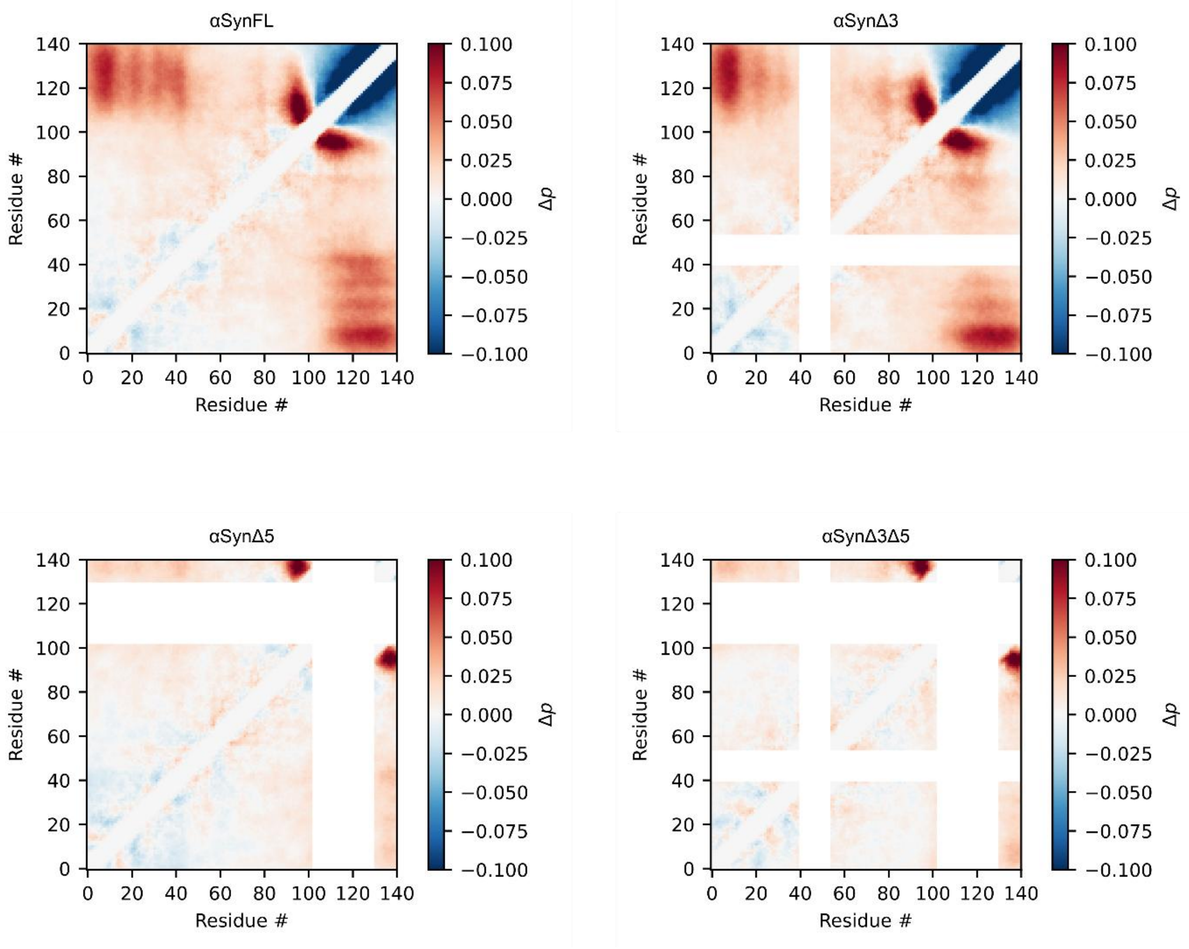

Figure S11. Contact probability difference maps for αSynFL, αSynΔ3, αSynΔ5, and αSynΔ3Δ5 at the lowest and highest ionic strength tested. Contact probability differences calculated by summing the contacts (<20 Å) for each residue pair at the lowest ionic strength (49 mM) and subtracting that of the highest ionic strength (453 mM), such that residue pairs with a positive (red) Δp are predicted to interact more frequently at low ionic strength, and negative (blue) Δp values represent residues that more frequently interact at higher ionic strengths.

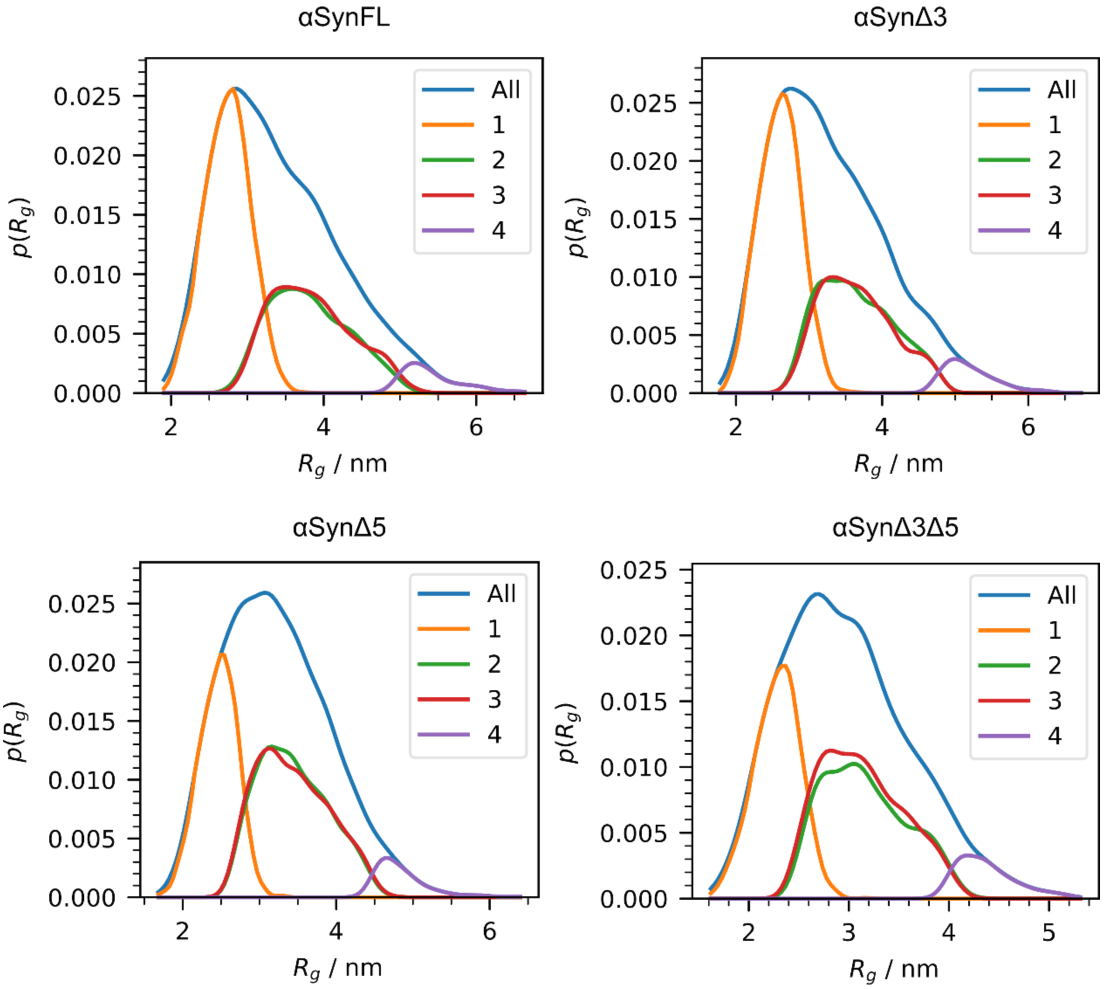

Figure S12. Spectral clustering of the alternative splice variants of αSyn. Probability distributions for the four alternative splice variants of αSyn from all 5,000 frames of the simulation and of each cluster. ‘1’, ‘2’, ‘3’, and ‘4’ refer to the distributions of R_g values for each of the four clusters resulting from the spectral clustering analysis (Figure 5).

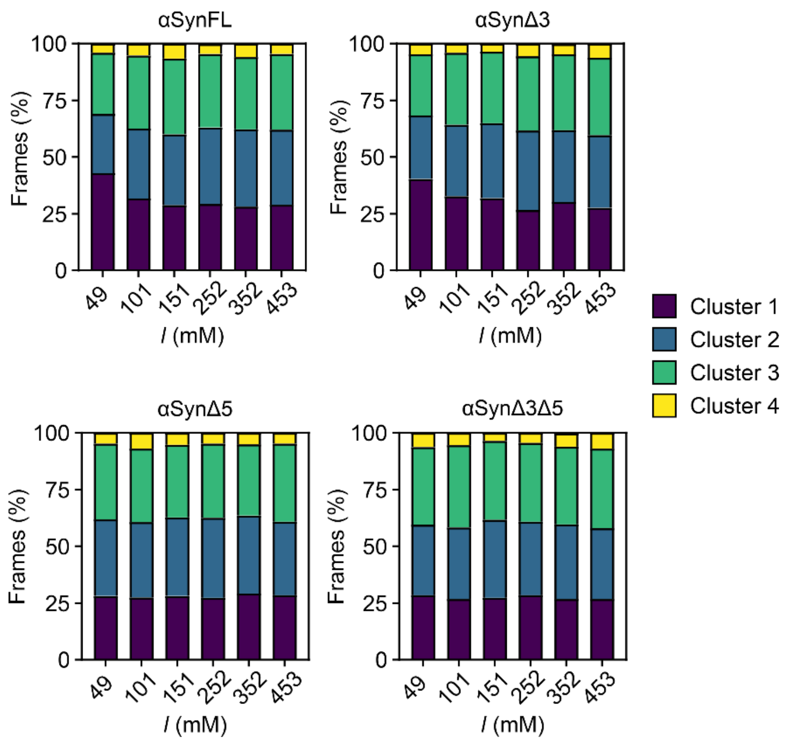

Figure S13. Percentage of frames in each cluster from the CALVADOS 2 simulations over a range of ionic strengths. Clusters are shown in the key. Note that I is the total ionic strength of the buffer, where 49 mM is equivalent to 20 mM sodium phosphate, 0 mM NaCl, at pH 7.4, and 453 mM is equivalent to 20 mM sodium phosphate, 400 mM NaCl at pH 7.4.

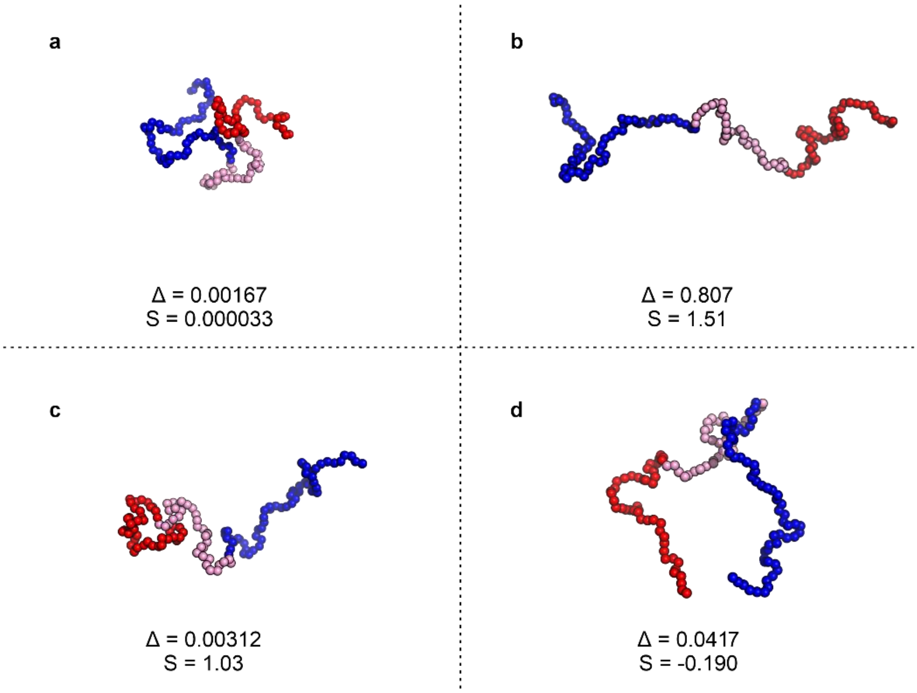

Figure S14. Representative conformations of αSynFL to demonstrate differences in Δ and S. (a) Example frame of αSynFL where both Δ and S values are close to 0, indicative of a spherical conformation. (b) Conformation in which both Δ and S are high, such that the protein is aspherical and highly prolate. (c) Conformation in which Δ is low but S is high, indicative of a prolate conformation with low asphericity. (d) Conformation where Δ is close to 0 but S is negative, indicating an oblate conformation.

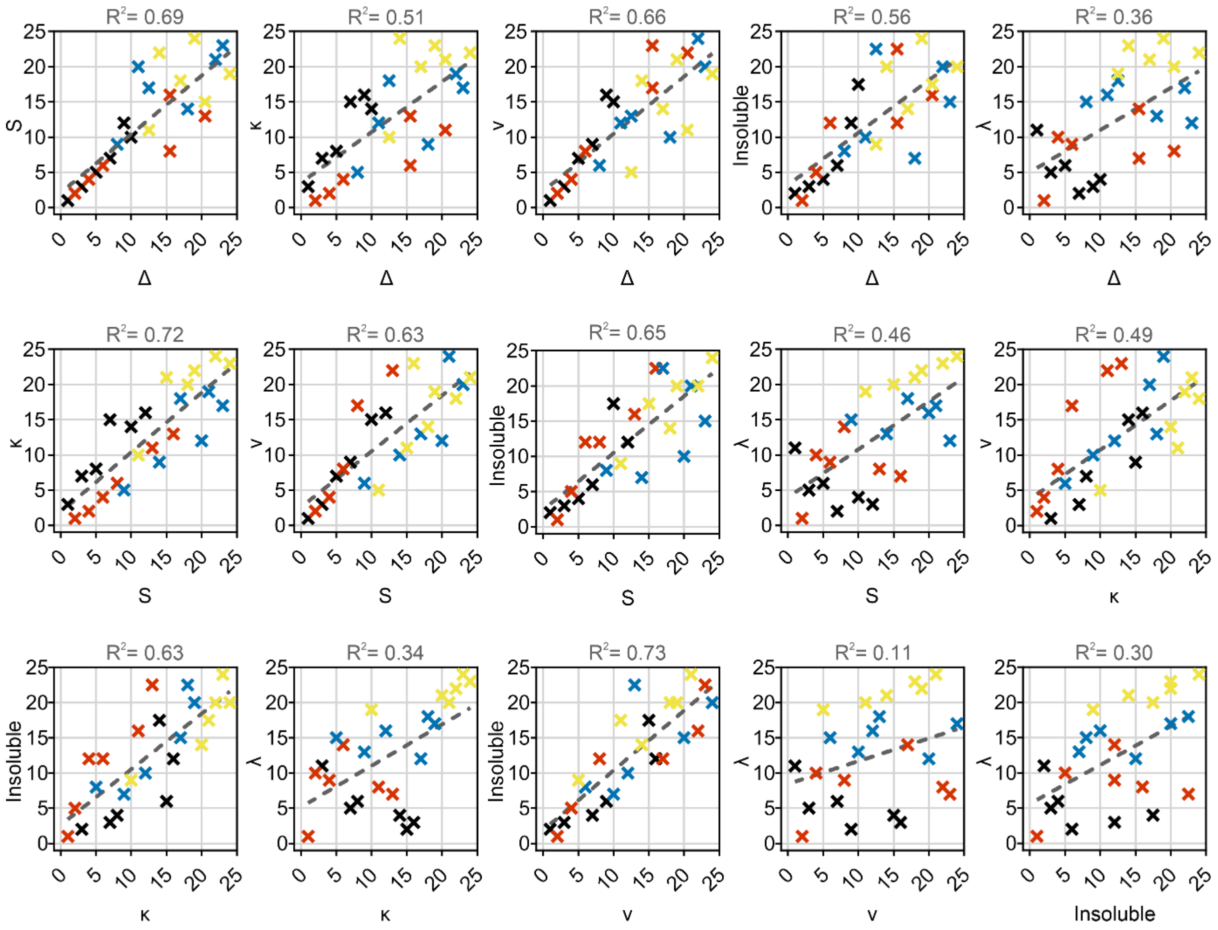

Figure S15. Correlations of the ranked data from the ionic strength dependent ThT assays and predictions from CALVADOS 2 simulations. Datapoints are coloured by variant, where αSynFL is black, αSynΔ3 is red, αSynΔ5 is blue, and αSynΔ3Δ5 is yellow. The six datapoints for each variant represent data from the six different ionic strengths tested here. Linear regression of the data is shown as the grey dashed line and the corresponding R^2 value (determined in GraphPad Prism 10.1.2) is displayed above each plot.

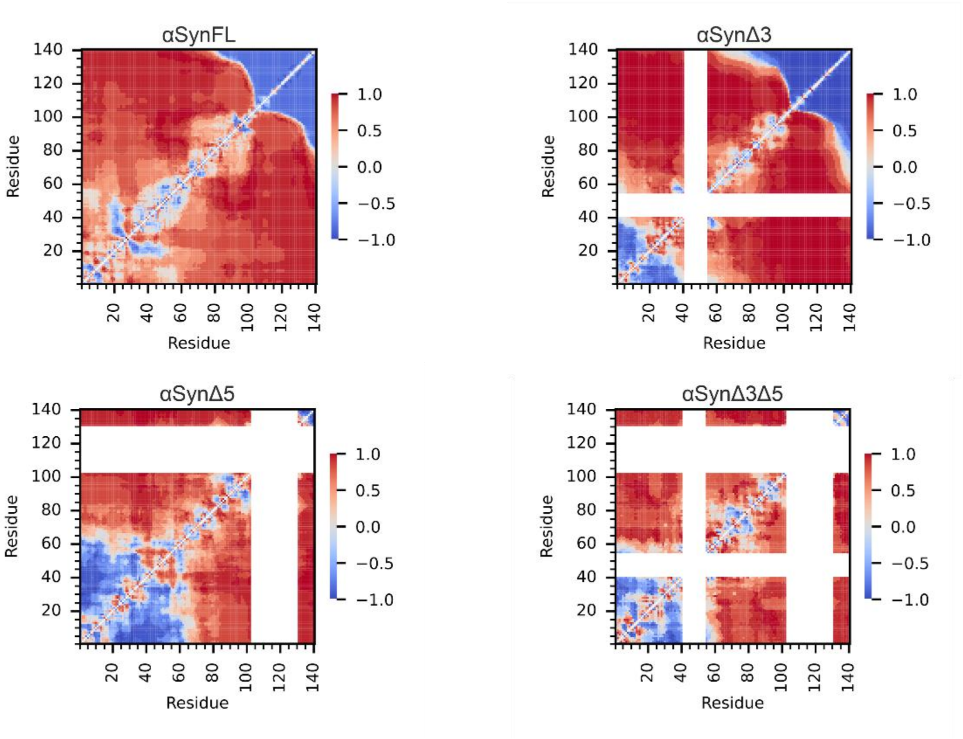

Figure S16. Spearman's rank heatmap of the inter-residue distances between all residue pairs of the alternative splice variants of αSyn from CALVADOS 2 simulations with experimentally derived κ values. Data shown for the individual variants of αSyn. Note that the residue pairs are padded for the variants missing exons, such that the residue identities are comparable.

Derivations

'Free Energy Barrier' model of the effect of ionic strength on κ

In this model, changes in κ with ionic strength reflect changes in the free energy barrier ($\Delta G^{\ddagger}$) for the dominant secondary process due to screening of electrostatic interactions by ions. The dominant secondary process can be secondary nucleation, in which the surface of an existing fibril templates the assembly of monomers to form a new fibril, or fragmentation, in which a fibril breaks in two to form separate fibrils capable of independent growth.

In the case of secondary nucleation, $\Delta G^{\ddagger}$ is the free energy required to form a critical nucleus, defined as the least stable species on the fibril formation pathway. The critical nucleus is most likely an oligomeric or small fibrillar aggregate on the surface of the existing catalytic amyloid fibril[13], although it could instead be a conformational transition state after dissociation of such a species from the fibril surface. In the case of fragmentation, $\Delta G^{\ddagger}$ is the free energy barrier for spontaneous breakage of a fibril[14].

If unfavourable electrostatics contribute to the free energy barrier for either process, then screening due to high ionic strength will reduce this barrier, and enhance the rate of the secondary pathway. Alternatively, if electrostatics are favourable and help to offset the free energy barrier, then screening will reduce the rate of the secondary pathway.

The macroscopic rate constant κ is equal to the root-product of the normalised rates of the dominant secondary process and elongation[15],

$$\kappa = \sqrt{2k_2 k_+ m(0)^{n_2+1}}, \qquad \text{[S1]}$$

where k_2 is the rate constant for the secondary process, k_+ is the rate constant for elongation, $m(0)$ is the initial free monomer concentration, and n_2 is the effective order of the secondary process ($n_2 > 0$ for secondary nucleation, $n_2 = 0$ for fragmentation).

The rate constant for the secondary process depends on the associated free energy barrier according to the Arrhenius equation,

$$k_2 = k_{2,0} e^{-\frac{\Delta G^{\ddagger}}{RT}}, \tag{S2}$$

where $k_{2,0}$ is the Arrhenius factor, R is the gas constant, and T is the temperature. Therefore,

$$\kappa = e^{-\frac{\Delta G^{\ddagger}}{2RT}} \sqrt{2k_{2,0}k_{+}m(0)^{n_2+1}}\ , \tag{S3}$$

We can further express $\Delta G^{\ddagger}$ as a sum of separate free energy terms due to non-electrostatic ($\Delta G^{\ddagger}_{\mathrm{nc}}$; nc for non-charged) and electrostatic ($\Delta G^{\ddagger}_{\mathrm{c}}$; c for charged) interactions,

$$\Delta G^{\ddagger} = \Delta G^{\ddagger}_{\mathrm{nc}} + \Delta G^{\ddagger}_{\mathrm{c}}\ . \tag{S4}$$

The strength of electrostatic interactions ($\Delta G^{\ddagger}_{\mathrm{c}}$) is expected to vary with the ionic strength, I, and this variation can be approximated by Debye-Hückel theory. In the simplified scenario where a single electrostatic interaction accounts for most of the variation in $\Delta G^{\ddagger}_{\mathrm{c}}$,

$$\Delta G^{\ddagger}_{\mathrm{c}} = \Delta G^{\ddagger}_{\mathrm{c},0} e^{-qr\sqrt{\frac{2I}{\varepsilon_r \varepsilon_0 k_B T}}}\ , \tag{S5}$$

where $\Delta G^{\ddagger}_{\mathrm{c},0}$ is the electrostatic free energy term without screening, r is the distance (or effective distance) between charged groups responsible for the interaction, q is the elementary charge, $\varepsilon_r \varepsilon_0$ is the permittivity of the solvent, and k_B is the Boltzmann constant.

Combining eq. [S3-S5] and rearranging, we obtain the result

$$\kappa = \exp\left(-\frac{\Delta G^{\ddagger}_{\mathrm{nc}} + \Delta G^{\ddagger}_{\mathrm{c},0} e^{-qr\sqrt{\frac{2I}{\varepsilon_r \varepsilon_0 k_B T}}}}{2RT} \right) \sqrt{2k_{2,0}k_{+}m(0)^{n_2+1}}\ , \tag{S6}$$

which predicts a sigmoidal variation of κ with ionic strength. By introducing identities for the lower limit, upper limit, and midpoint of this sigmoid, we can simplify the expression,

$$\kappa = \kappa_{\mathrm{sat}} \left(\frac{\kappa_0}{\kappa_{\mathrm{sat}}}\right)^{2^{-\sqrt{I/I_{\mathrm{mid}}}}} . \quad \text{[S7]}$$

Here, κ_0 and κ_{sat} are the low- and high-ionic strength limits of κ at which there is either no screening or complete screening of the electrostatic component of the nucleation free energy barrier, respectively. These have the definitions,

$$\kappa_0 = e^{-\frac{\Delta G^{\ddagger}_{\mathrm{nc}} + \Delta G^{\ddagger}_{\mathrm{c},0}}{2RT}} \sqrt{2k_{2,0}k_+ m(0)^{n_2+1}} , \quad \text{[S8]}$$

$$\kappa_{\mathrm{sat}} = e^{-\frac{\Delta G^{\ddagger}_{\mathrm{nc}}}{2RT}} \sqrt{2k_{2,0}k_+ m(0)^{n_2+1}} . \quad \text{[S9]}$$

If the electrostatics are unfavourable ($\Delta G^{\ddagger}_{\mathrm{c},0} > 0$), κ_0 will be lower than κ_{sat}, and vice versa. I_{mid} is the ionic strength at which κ is half-way between κ_0 and κ_{sat} on a logarithmic scale, i.e. $\log \kappa = (\log \kappa_0 + \log \kappa_{\mathrm{sat}})/2$, and has the definition,

$$I_{\mathrm{mid}} = \frac{\varepsilon_r \varepsilon_0 k_B T}{2} \left(\frac{\ln 2}{qr}\right)^2 . \quad \text{[S10]}$$

Thus, I_{mid} is primarily determined by the effective range r of the electrostatics that are being screened; the closer the interaction, the higher the ionic strength that will be required to screen it out. The screening transition will occur when the Debye screening length λ_D becomes approximately less than r, so that the electrostatic term in equation [S4] is screened out.

In practice, it was not possible to use sufficiently low ionic strengths to observe the limit of κ at low ionic strength, so we did not attempt to analyse $\Delta G^{\ddagger}_{\mathrm{c},0}$ or r. However, we were able to observe the limit of κ at high ionic strength for all variants except for αSynΔ3, which provided sufficient data to perform a model comparison with the Brønsted-Bjerrum model, and determine $\Delta\Delta G^{\ddagger}_{\mathrm{nc}}$ based on fold-changes in κ_{sat} according to equation [S9].

'Brønsted-Bjerrum' model of the effect of ionic strength on κ

In this model, which is only compatible with a multimolecular process such as secondary nucleation, changes in κ are due to the differing effect of ionic strength on the activity of the precursors of secondary nucleation (monomer and fibrils) and an intermediate state (monomers associated with fibril). A model of this sort was previously assumed by Meisl *et al.*[16] in their analysis of the effects of ionic strength.

Suppose secondary nucleation proceeds via an activated intermediate $m_n{:}\,F$, which exists in equilibrium with monomer M and fibril F.

$$nM + F \leftrightharpoons m_n{:}\,F \longrightarrow 2F \quad \text{[S11]}$$

In this case, the generalised Brønsted-Bjerrum equation is

$$\ln k_2 = \ln k'_{2,0} + Anz_m(nz_m + 2z_F - z_m)\sqrt{I}\,, \quad \text{[S12]}$$

where $k'_{2,0}$ is the rate constant for secondary nucleation at zero ionic strength, A is a constant whose precise value depends on the solvent and temperature but is approximately 0.5 $\mathrm{M}^{-1/2}$, z_m is the charge of the monomer, and z_F is the effective charge of the binding site on the fibril.

Combining eq. [S12] with eq. [S1] and expressing in terms of κ_0 and I_2,

$$\kappa = \kappa_0 2^{\sqrt{\frac{I}{I_2}}}\,, \quad \text{[S13]}$$

where κ_0 is the limit of κ at low ionic strength,

$$\kappa_0 = \sqrt{2k'_{2,0}k_+m(0)^{n_2+1}}\,, \quad \text{[S14]}$$

and I_2 is the ionic strength at which there is a two-fold enhancement of κ,

$$I_2 = \left[\frac{2\ln 2}{Anz_m(nz_m+2z_F-z_m)}\right]^2. \quad \text{[S15]}$$

Note that eq. [S13] has no upper limit. Unlike the ‘Free Energy Barrier’ model, the ‘Brønsted-Bjerrum’ model predicts an unrestricted enhancement of κ as ionic strength increases.

References

(1) Beyer, K.; Lao, J. I.; Carrato, C.; Mate, J. L.; López, D.; Ferrer, I.; Ariza, A. Differential Expression of α-Synuclein Isoforms in Dementia with Lewy Bodies. *Neuropathology and Applied Neurobiology* **2004**, *30* (6), 601–607. https://doi.org/10.1111/j.1365-2990.2004.00572.x.

(2) Beyer, K.; Humbert, J.; Ferrer, A.; Lao, J. I.; Carrato, C.; López, D.; Ferrer, I.; Ariza, A. Low Alpha-Synuclein 126 mRNA Levels in Dementia with Lewy Bodies and Alzheimer Disease. *NeuroReport* **2006**, *17* (12), 1327. https://doi.org/10.1097/01.wnr.0000224773.66904.e7.

(3) Beyer, K.; Domingo-Sàbat, M.; Humbert, J.; Carrato, C.; Ferrer, I.; Ariza, A. Differential Expression of Alpha-Synuclein, Parkin, and Synphilin-1 Isoforms in Lewy Body Disease. *Neurogenetics* **2008**, *9* (3), 163–172. https://doi.org/10.1007/s10048-008-0124-6.

(4) McLean, J. R.; Hallett, P. J.; Cooper, O.; Stanley, M.; Isacson, O. Transcript Expression Levels of Full-Length Alpha-Synuclein and Its Three Alternatively Spliced Variants in Parkinson's Disease Brain Regions and in a Transgenic Mouse Model of Alpha-Synuclein Overexpression. *Mol Cell Neurosci* **2012**, *49* (2), 230–239. https://doi.org/10.1016/j.mcn.2011.11.006.

(5) Cardo, L. F.; Coto, E.; de Mena, L.; Ribacoba, R.; Mata, I. F.; Menéndez, M.; Moris, G.; Alvarez, V. Alpha-Synuclein Transcript Isoforms in Three Different Brain Regions from Parkinson's Disease and Healthy Subjects in Relation to the *SNCA* Rs356165/Rs11931074 Polymorphisms. *Neuroscience Letters* **2014**, *562*, 45–49. https://doi.org/10.1016/j.neulet.2014.01.009.

(6) Brudek, T.; Winge, K.; Rasmussen, N. B.; Bahl, J. M. C.; Tanassi, J.; Agander, T. K.; Hyde, T. M.; Pakkenberg, B. Altered α-Synuclein, Parkin, and Synphilin Isoform Levels in Multiple System Atrophy Brains. *Journal of Neurochemistry* **2016**, *136* (1), 172–185. https://doi.org/10.1111/jnc.13392.

(7) Meisl, G.; Kirkegaard, J. B.; Arosio, P.; Michaels, T. C. T.; Vendruscolo, M.; Dobson, C. M.; Linse, S.; Knowles, T. P. J. Molecular Mechanisms of Protein Aggregation from Global Fitting of Kinetic Models. *Nat Protoc* **2016**, *11* (2), 252–272. https://doi.org/10.1038/nprot.2016.010.

(8) Zheng, W.; Dignon, G.; Brown, M.; Kim, Y. C.; Mittal, J. Hydropathy Patterning Complements Charge Patterning to Describe Conformational Preferences of Disordered Proteins. *J Phys Chem Lett* **2020**, *11* (9), 3408–3415. https://doi.org/10.1021/acs.jpclett.0c00288.

(9) Sawle, L.; Ghosh, K. A Theoretical Method to Compute Sequence Dependent Configurational Properties in Charged Polymers and Proteins. *J Chem Phys* **2015**, *143* (8), 085101. https://doi.org/10.1063/1.4929391.

(10) Das, R. K.; Pappu, R. V. Conformations of Intrinsically Disordered Proteins Are Influenced by Linear Sequence Distributions of Oppositely Charged Residues. *Proc Natl Acad Sci U S A* **2013**, *110* (33), 13392–13397. https://doi.org/10.1073/pnas.1304749110.

(11) Wilkins, D. K.; Grimshaw, S. B.; Receveur, V.; Dobson, C. M.; Jones, J. A.; Smith, L. J. Hydrodynamic Radii of Native and Denatured Proteins Measured by Pulse Field Gradient NMR Techniques. *Biochemistry* **1999**, *38* (50), 16424–16431. https://doi.org/10.1021/bi991765q.

(12) Michaels, T. C. T.; Dear, A. J.; Knowles, T. P. J. Universality of Filamentous Aggregation Phenomena. *Phys. Rev. E* **2019**, *99* (6), 062415. https://doi.org/10.1103/PhysRevE.99.062415.

(13) Cohen, S. I. A.; Cukalevski, R.; Michaels, T. C. T.; Šarić, A.; Törnquist, M.; Vendruscolo, M.; Dobson, C. M.; Buell, A. K.; Knowles, T. P. J.; Linse, S. Distinct Thermodynamic Signatures of Oligomer Generation in the Aggregation of the Amyloid-β Peptide. *Nature Chem* **2018**, *10* (5), 523–531. https://doi.org/10.1038/s41557-018-0023-x.

(14) Zaccone, A.; Terentjev, I.; Herling, T. W.; Knowles, T. P. J.; Aleksandrova, A.; Terentjev, E. M. Kinetics of Fragmentation and Dissociation of Two-Strand Protein Filaments: Coarse-Grained Simulations and Experiments. *J. Chem. Phys.* **2016**, *145* (10). https://doi.org/10.1063/1.4962366.

(15) Cohen, S. I. A.; Vendruscolo, M.; Dobson, C. M.; Knowles, T. P. J. Nucleated Polymerization with Secondary Pathways II. Determination of Self-Consistent Solutions to Growth Processes Described by Non-Linear Master Equations. *J Chem Phys* **2011**, *135* (6), 065106. https://doi.org/10.1063/1.3608917.

(16) Meisl, G.; Yang, X.; Dobson, C. M.; Linse, S.; Knowles, T. P. J. Modulation of Electrostatic Interactions to Reveal a Reaction Network Unifying the Aggregation Behaviour of the Aβ42 Peptide and Its Variants. *Chem. Sci.* **2017**, *8* (6), 4352–4362. https://doi.org/10.1039/C7SC00215G.
